# Anabolic actions of PTH in murine models: two decades of insights

**DOI:** 10.1002/jbmr.4389

**Published:** 2021-07-27

**Authors:** Laura E. Zweifler, Amy J. Koh, Stephanie Daignault-Newton, Laurie K. McCauley

**Affiliations:** 1 Department of Periodontics and Oral Medicine, University of Michigan School of Dentistry, Ann Arbor, Michigan, USA; 2 Biostatistics Unit, University of Michigan School of Public Health, Ann Arbor, Michigan, USA; 3 Department of Pathology, Medical School, University of Michigan, Ann Arbor, Michigan, USA

**Keywords:** ANABOLIC, BONE ANABOLISM, GENETIC ANIMAL MODELS, PARATHYROID-RELATED DISORDERS, PTH

## Abstract

Parathyroid hormone (PTH) is produced by the parathyroid glands in response to low serum calcium concentrations where it targets bones, kidneys, and indirectly, intestines. The N-terminus of PTH has been investigated for decades for its ability to stimulate bone formation when administered intermittently (iPTH) and is used clinically as an effective anabolic agent for the treatment of osteoporosis. Despite great interest in iPTH and its clinical use, the mechanisms of PTH action remain complicated and not fully defined. More than 70 gene targets in more than 90 murine models have been utilized to better understand PTH anabolic actions. Because murine studies utilized wild-type mice as positive controls, a variety of variables were analyzed to better understand the optimal conditions under which iPTH functions. The greatest responses to iPTH were in male mice, with treatment starting later than 12 weeks of age, a treatment duration lasting 5–6 weeks, and a PTH dose of 30–60 μg/kg/day. This comprehensive study also evaluated these genetic models relative to the bone formative actions with a primary focus on the trabecular compartment revealing trends in critical genes and gene families relevant for PTH anabolic actions. The summation of these data revealed the gene deletions with the greatest increase in trabecular bone volume in response to iPTH. These included PTH and 1-α-hydroxylase (*Pth;1α(OH)ase*, 62-fold), amphiregulin (*Areg*, 15.8-fold), and PTH related protein (*Pthrp*, 10.2-fold). The deletions with the greatest inhibition of the anabolic response include deletions of: proteoglycan 4 (*Prg4*, −9.7-fold), low-density lipoprotein receptor-related protein 6 (Lrp6, 1.3-fold), and low-density lipoprotein receptor-related protein 5 (*Lrp5*, −1.0-fold). Anabolic actions of iPTH were broadly affected via multiple and diverse genes. This data provides critical insight for future research and development, as well as application to human therapeutics.

## INTRODUCTION

Parathyroid hormone (PTH) has been approved by the US Food and Drug Administration (FDA) since 2002, when teriparatide, a 34–amino acid analog of PTH, was accepted for the treatment of osteoporosis. More recently a PTH related protein (PTHrP) analog was also approved for the treatment of osteoporosis under the name abaloparatide.^(1)^ It is well accepted that intermittent PTH (iPTH) therapy is anabolic for bone, whereas continuous PTH exposure is catabolic. The anabolic actions of iPTH in bone have been observed in animal models since 1929 using cats and rats.^(2–5)^ These results were recapitulated in human patients,^(6,7)^ which led to the approval of this anabolic agent for therapeutic purposes. However, the anabolic mechanism of iPTH is not fully understood, and this study aimed to reveal trends in critical genes and gene families relevant for iPTH anabolic actions.

As an endogenous endocrine mediator, PTH is released when the parathyroid gland detects a decrease in serum calcium concentration. Circulating PTH then targets the kidney and bone to increase serum calcium levels.^(5)^ The effects of PTH and PTHrP in bone are achieved by binding to its type 1 receptor (PTH1R, a G-protein coupled receptor with seven transmembrane domains) on osteoblasts.^(8,9)^ This stimulates the production of receptor activator of nuclear factor κB ligand (RANKL) in osteoblasts and subsequent osteoclastogenesis.^(10)^ Indirectly, there is an increase in osteoblast numbers and bone formation.^(11)^

PTH is essential for fetal development, with newborn PTH-deficient mice exhibiting reduced cartilage matrix mineralization and trabecular bone, due to fewer metaphyseal osteoblasts.^(12)^ Adult PTH-null mice exhibit decreased serum calcium, decreased 1,25-dihydroxyvitamin D_3_, and increased serum phosphate.^(13)^ Trabecular bone volume is increased in the femurs, tibias, and vertebrae of mutant mice, and the number and size of tibial osteoclasts are reduced. Furthermore, there is a decreased mineral apposition rate.

PTHrP-null mice exhibit an osteoporotic phenotype that can be recapitulated in mice with targeted deletion in osteoblasts (*Pthrp*^*f/f*^*;cre*^*colI*^).^(14)^ This model is more specific to the local bone environment, in which iPTH treatment increased mineral apposition rate, bone volume, trabecular number, trabecular thickness, trabecular connectivity, and cortical thickness in long bones. This could be attributed to increased receptor availability without endogenous PTHrP or changes in receptor desensitization (i.e., increased number of receptors because there is not desensitization from PTHrP). In either case, it is likely that PTHrP can modulate the response to PTH via the PTH1R receptor.^(14)^

## MATERIALS AND METHODS

**FIGURE 1.**
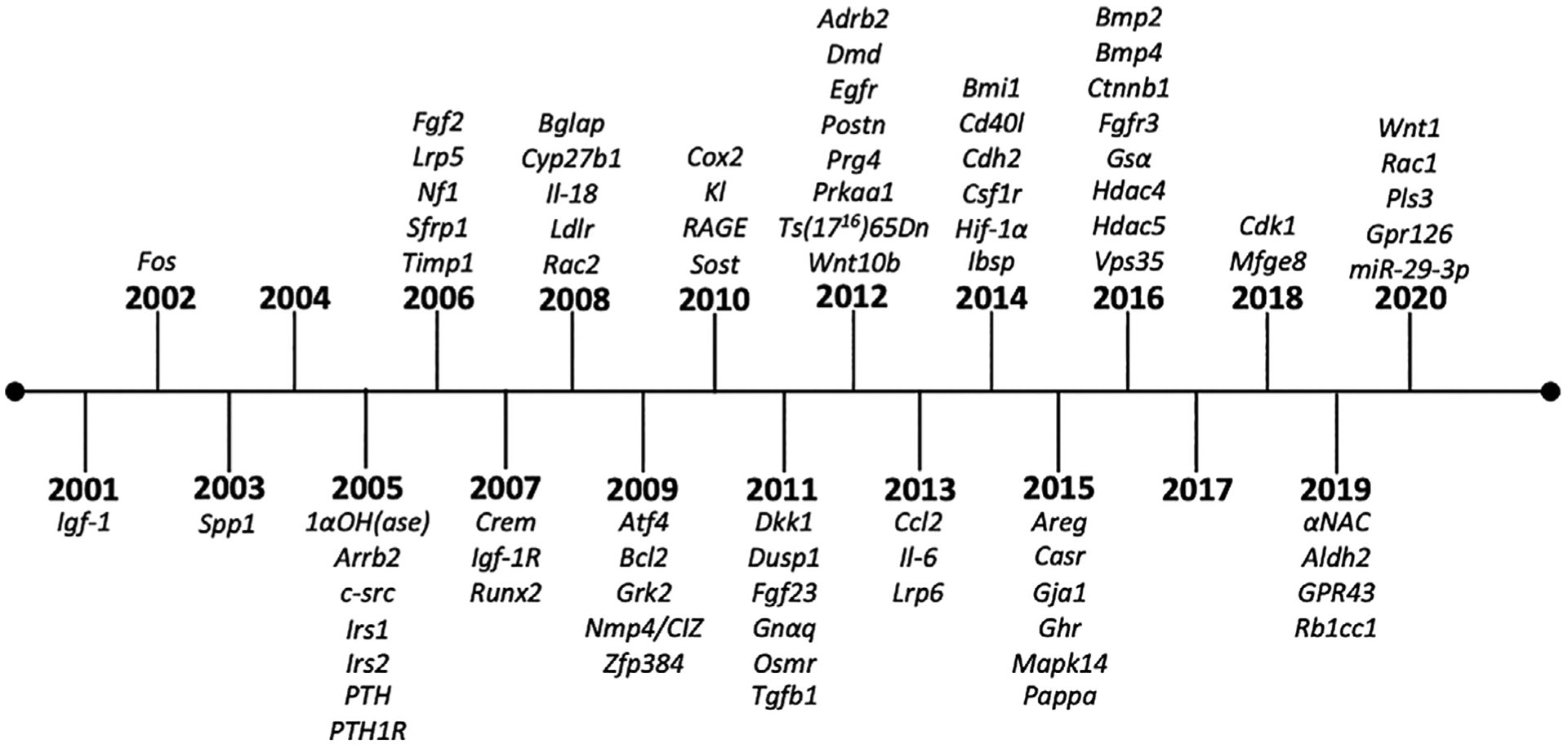
Timeline of gene targeted mouse models of PTH anabolic actions in bone. Abbreviation: PTH, parathyroid hormone.

Data for this study was collected from publications that have administered anabolic doses of iPTH from 2001 to 2020 (Figure 1). Papers were accessed by searching scholarly search engines, such as PubMed, through December 2020. A highly relevant and consistent outcome of trabecular bone volume per total volume was used as a key and focused measure to compare the anabolic response in experimental gene targeted mice to wild-type controls in published studies. The PTH-induced bone volume response was derived for both gene targeted and wild-type mice (Table 1) separately [(PTH – Vehicle)/PTH]. Then, the relative response was calculated as a fold change by dividing the gene targeted response by the wild-type response. A fold change of 1.0 indicates that there was no change in the anabolic response between wild-type and gene-targeted mice. If the fold change was greater than 1.0, the mutant mice had a greater anabolic response than wild-type mice, whereas between 0 and 1.0 the mutant mice had a less anabolic response. A negative fold change indicates that the mutant response to iPTH was not anabolic. In some studies, actual numerical data was provided, whereas in others, data was derived from graphic representation. When bone volume was only depicted graphically, values were estimated by measurement with a ruler to derive the gene-targeted response relative to wild type. Studies that showed an anabolic response to PTH in wild-type controls were included whereas those that did not demonstrate an anabolic response in controls were excluded (there were very few studies that did not display an anabolic response).

Most commonly, human PTH(1–34) (hPTH(1–34)) was administered, although there were a few studies as indicated when the PTH differed (i.e., hPTH(1–84) or derived from a different source). Doses ranged from 20 to 160 μg/kg/day, but was typically between 40 and 100 μg/kg/day, as specified in Table 2. PTH was administered by injection daily, 7 days/week, unless noted differently. Treatment time was typically 2 to 6 weeks of iPTH. The models are grouped under categories largely according to functional analyses in the Supplemental Material, alphabetically in Table 2. By assimilating the literature that has used anabolic PTH in genetic mouse models, we gain a better understanding of key genetic pathways as well as the overall complexity of PTH actions in bone.

## RESULTS

### Actions of iPTH in wild-type mice

**FIGURE 2.**
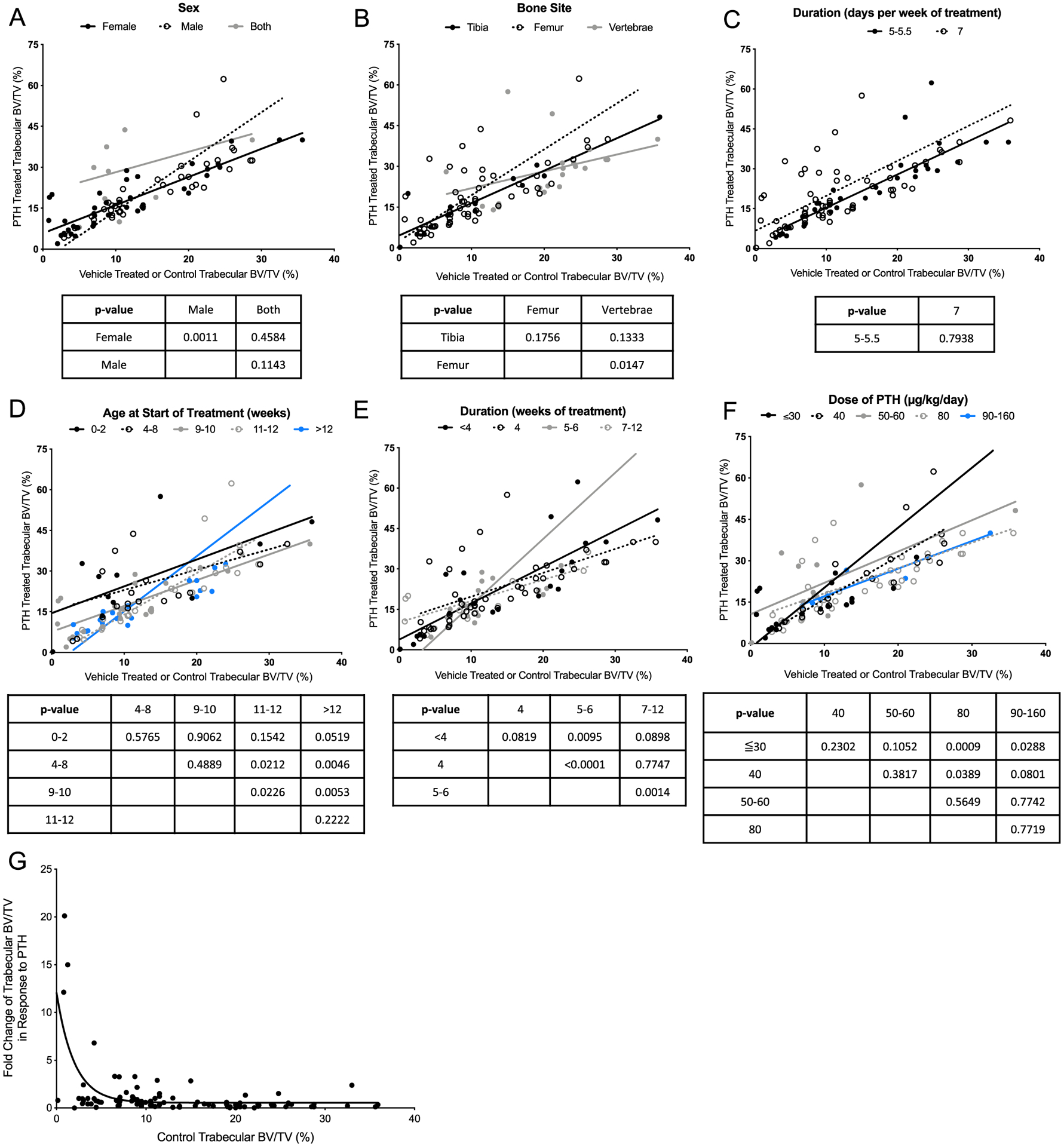
Trabecular bone response in WT mice. (*A*–*F*) Trabecular bone volume is graphed for vehicle-treated (*x* axis) and PTH-treated (*y* axis) WT mice. Each plot stratifies a different variable, including (*A*) sex, (*B*) bone site analyzed, (*C*) duration (days per week of treatment), (*D*) age at the start of treatment, (*E*) duration (weeks of treatment), or (*F*) dose of treatment. Linear regression of the slope was analyzed for each group and compared within a variable. The *p* values are reported in the charts under each graph, and correspond to the analysis between the column and row headers (i.e., in (A), the slope of the line for male and female has a p-value of 0.0011). (*G*) Control trabecular bone volume in WT mice and the FC of trabecular bone volume in response to PTH in WT mice is plotted. The AIC is a statistical predictor of error between two models, and was used to confirm an inverse exponential relationship between control bone volume and the FC in bone volume with PTH in WT mice. Abbreviations: AIC, Akaike Information Criterion; PTH, parathyroid hormone; WT, wild-type; FC, fold change.

Because gene-targeted murine studies utilized wild-type mice as positive controls, a variety of variables were analyzed to better understand the optimal conditions under which iPTH functions. Trabecular bone volume was compiled and organized by different categories (Figure 2, Table 1). The groups were stratified by: sex, bone site, days per week of treatment, age at start of treatment, duration of treatment, and dose of iPTH. Strain was also considered and is listed in Table 2; however, the only strain that had a large enough sample size for consideration was C57BL/6. Because the interest of this section is in comparing different categories, we did not include strain in the analysis. Most of these groups had a significant, positive correlation between the control trabecular bone volume and the iPTH-treated bone volume (Table 1). Using both sexes was an exception. Although this does not suggest that those indices should not be used in future studies, caution should be taken if drawing conclusions based only on trabecular bone volume.

Correlation graphs of the reported trabecular bone volume in control versus iPTH mice are shown in Figure 2 and are separated by the categories mentioned. In order to understand how the variables relate within a category, the data was modeled with a linear regression and the slopes and corresponding 95% confidence interval were compared. Groups that had a significant correlation are discussed in the Supplemental Material, but all of the data is presented. This data can be used to inform future study design and interpretation.

We hypothesized that if a mouse has a high baseline bone volume, there is less capacity to mount an anabolic response to iPTH. Similarly, if an animal has a low baseline bone volume, they would show a greater response to iPTH. Analysis of the graph in Figure 2G supports this, with the control bone volume plotted against the fold change response to PTH. Although biases exist because only studies that showed an anabolic response in wild-type mice were included, statistics support an inverse exponential relationship between these variables. To confirm that the data had an exponential relationship, and not a linear one, we calculated the Akaike Information Criterion (AIC), a statistical predictor of error between two models. The AIC for the exponential model is 36.44 lower than the linear model, indicating that the exponential equation more precisely describes the relationship between the two variables.

### Analysis of PTH anabolic actions in bone using gene-targeted mice

**FIGURE 3.**
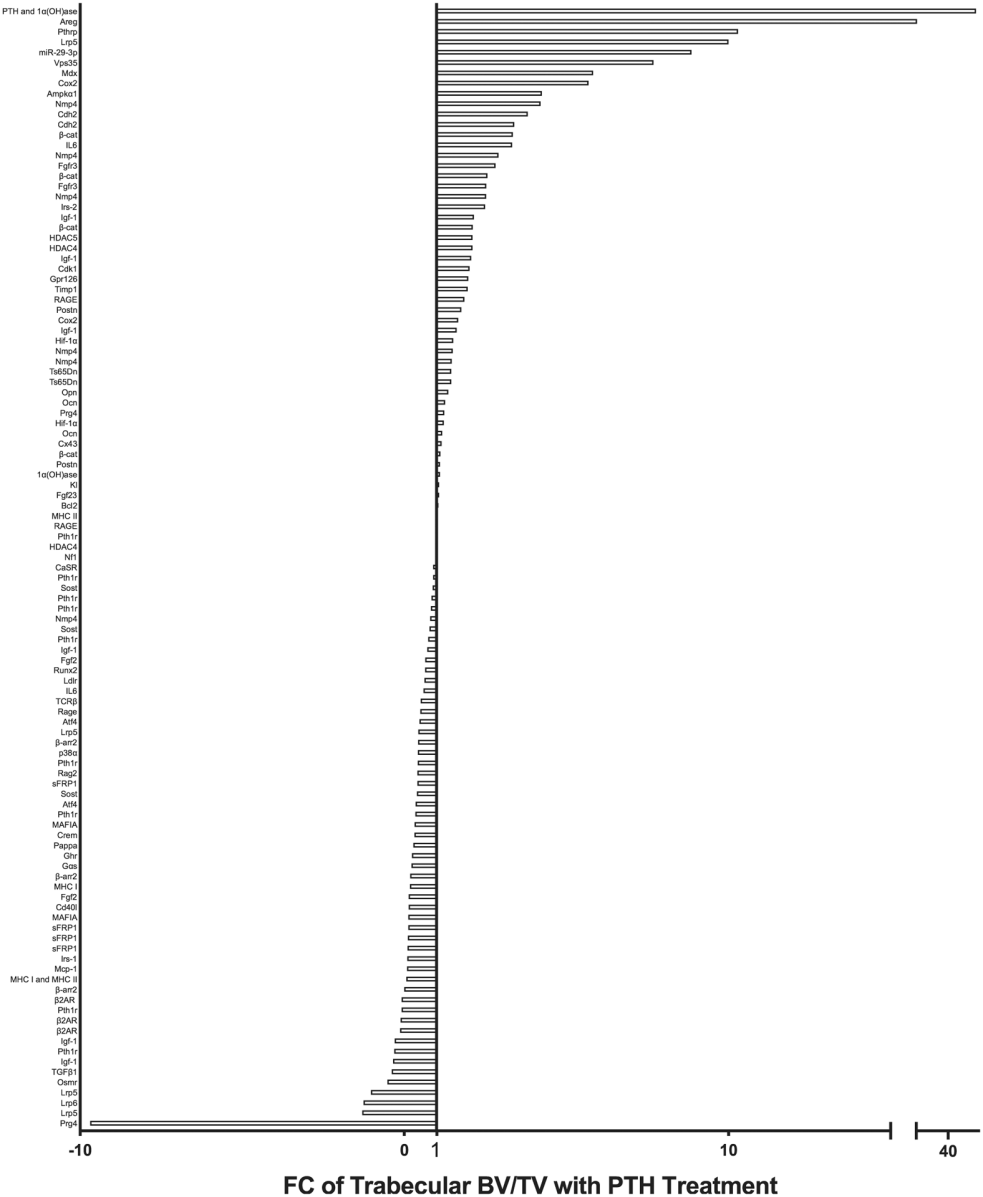
FC of PTH-/control-treated trabecular bone volume per total volume per targeted gene model. The response to PTH treatment in gene targeted murine models was calculated using the bone volume FC in mutant mice relative to the FC of control treated mice. The x axis lists the targeted gene. Some genes are listed multiple times, each of which represents a different study or cohort of animals listed in Table 2. If there was no change between control and genetically modified treated animals, the FC is 1, indicated by the marked line. Abbreviations: FC, fold change; PTH, parathyroid hormone.

The mechanism of anabolic iPTH and its effect on the bone microenvironment has been studied for decades, and numerous mechanisms have been proposed based on in vitro and in vivo models.^(67–69)^ A wide variety of genetic mouse models have been employed to elucidate the actions of PTH in bone over the past 20 years (Figures 1, 3, Table 2). With modern technology facilitating unprecedented genetic manipulation, this comprehensive study compiles the evidence of iPTH actions in gene-targeted murine models. Of note, an important limitation is that although some mutations are global, many are focused on a subset of cells, and dependent on effective cre drivers and appropriate promoter selection. Hence the anabolic actions of PTH may reflect the effectiveness of the model as well as the targeted gene. Specific genotypes are indicated in Table 2, and are discussed in detail in the Supplemental Materials.

The Supplemental Materials include detailed text descriptions of the literature using iPTH in gene-targeted mice, which are summarized alphabetically by gene in Table 2. The models studied can be stratified by the function of the gene, including receptor activation and signaling pathways; downstream mediators in the fibroblast growth factor (FGF) family, wingless-related integration site (Wnt) family, bone morphogenetic protein (BMP) family, insulin-like growth factor (IGF), and growth hormone (GH), epidermal growth factor (EGF) family; and cell regulatory factors including apoptotic, immunity, extracellular matrix (ECM), cytoskeletal, and calcium regulation. The summation of these data demonstrated the gene deletions with the greatest increase in response to iPTH. These included PTH and 1-α-hydroxylase (*Pth;1α(OH)ase*, 62-fold)^(70)^, amphiregulin (*Areg*, 15.8-fold),^(17)^ and PTH-related protein (*Pthrp*, 10.2-fold).^(14)^ (Table 2). The deletions with the greatest inhibition of the anabolic response include deletions of: proteoglycan 4 (*Prg4*, −9.7-fold),^(71)^ low-density lipoprotein receptor-related protein 6 (*Lrp6*, 1.3-fold),^(64)^ and low-density lipoprotein receptor-related protein 5 (*Lrp5*, −1.0-fold)^(63)^ (Table 2). Several notable genes demonstrated no alteration of the anabolic action of PTH, including major histocompatibility complex II knockout mice (*Mhc II*),^(72)^ bone sialoprotein (*Bsp*),^(28)^ and histone deacetylase 4 (*Hdac4*).^(50)^ The models with the most study were insulin-like growth factor-1 (*Igf-1*).^(52–55)^

By detailing comparisons between reported iPTH studies, we are able to assimilate the role of different genes in the anabolic response. For example, Table 2 shows that mice with mutations in *Igf-1* can range in their response to iPTH, with bone volume fold changes relative to control mice from −0.3-fold to 2.1-fold.^(52–56)^ There has been long-standing interest in this gene; it was the first genetic model to be studied with iPTH in 2001 because of the increase in IGF-1 production from osteoblasts in response to PTH.^(52)^ A detailed analysis in the Supplemental Material compares the study design, mouse genetics, and conclusions of each report. These studies support a necessary role of IGF-1 in the anabolic response, as well as downstream targets, such as insulin receptor substrate-1 (IRS-1).^(59)^

## DISCUSSION

When mice are administered anabolic doses of PTH, signaling cascades affect proliferation and development of osteoblasts. There are many protein interactions and regulatory factors involved in this process, and it is unsurprising that when they are disrupted, the anabolic response does not achieve its full potential. The purpose of this study was to further elucidate PTH mechanisms by collectively analyzing the extensive work performed using mouse models.

The anabolic response in wild-type mice was analyzed to understand baseline differences and influences. Of the variables analyzed, the greatest responses to iPTH were in male mice, with treatment starting later than 12 weeks of age, a treatment duration lasting 5 to 6 weeks, and a PTH dose of 30 to 60 μg/kg/day. This data should be used to inform future study design for efficient use of resources. For example, based on the correlation data, male and female mice should be analyzed separately when treated with iPTH.

Collectively, the data suggests that starting treatment at greater than 12 weeks of age yields the highest response to iPTH. Mice are considered mature adults at this stage, but peak bone mass is closer to 16 to 18 weeks. The murine skeleton continues to grow past sexual maturity (about 7 weeks), whereas the human skeleton does not. PTH is commonly prescribed in postmenopausal women, and this population would be more comparative to mice that are at least 12 months old. Of the more than 130 cohorts of mice studied, only one was in this age range.^(25)^

Administering PTH for at least 5 days per week is sufficient to yield an anabolic response. Although it is well documented that whereas continuous PTH is catabolic, iPTH is anabolic,^(73)^ this analysis has focused on the anabolic studies. Frolik et al.^(74)^ used a rat model to determine that the pharmacokinetics of PTH(1–34) varies with differing treatment regimens. They found giving the same 80 μg/kg of PTH in a single injection or via six injections over 1 h resulted in an anabolic response. However, administering the same 80 μg/kg of PTH over 6 or 8 h produced a catabolic response. They associated the anabolic iPTH in a temporal manner with the rapid increase in serum calcium, followed by tapering.

Analyses for this examination focused on the tibias, femurs, and vertebrae. Although studies analyzing calvariae are reported in Table 2, there were not enough to include in the correlation analysis. In humans, bone mineral density in postmenopausal women that were randomly assigned to PTH or placebo showed a larger percent change in the lumbar spine than femoral neck.^(7)^ Of note, this is comparing different outcomes (bone volume for murine studies and bone mineral density for human), measured by different variables, and in a quadrupedal versus a bipedal species.

Relative to specific genetic aberrations that may inform PTH mechanisms, several trends are apparent from this analysis of more than 90 gene-targeted studies. Bone health and energy metabolism are linked formulating a vital area of research interest. Many clinical conditions are also linked to altered energy expenditure, as reviewed by Motyl et al.^(75)^ Among these targeted murine models with the largest increases in anabolic response to iPTH were AMP-activated protein kinase α1 (Ampkα1), hypoxia-inducible factor 1-alpha (Hif-1α), and cyclooxygenase-2 (Cox2). Ampkα1 regulates energy consumption in the cell, working to promote adenosine triphosphate (ATP) conservation or expenditure depending on current conditions.^(76)^ Mice lacking Ampkα1 have a low bone mass with an increased anabolic response to iPTH.^(16)^ Hif-1α is referred to as the master regulator of hypoxia because it is an oxygen-sensitive subunit of the Hif-1 complex (with Hif-1β). When oxygen is not present, Hif-1α is stabilized and translocated to the nucleus to bind to hypoxia-response elements.^(77)^ Cox2 has been identified as a hypoxia responsive gene in colorectal cancer.^(78)^ Authors of the work with Cox2 and iPTH were interested in its role regulating prostaglandin production, but it is possible that part of the effect of deleting this gene is affected by changes in energy metabolism. When these genes are deleted, the responsiveness to iPTH in bone is enhanced. Because these genes are activated when the cell is under metabolic stress and their actions limit the PTH response, it is conceivable that they allow the cell to work at the capacity allowed by current energy conditions, limited by oxygen concentrations.

Ampkα1 and Hif-1α both regulate autophagy.^(79,80)^ PTH prevents osteoblast apoptosis, prolonging the life of these cells.^(81)^ It is also possible that in the absence of these genes, cell survival is further enhanced, leading to an increased response to iPTH. A presentation at the American Society for Bone and Mineral Research Annual Meeting in 2019 further connected autophagy and PTH mechanisms.^(82)^ Using mice that had autophagydeficient osteoblasts (*Fip200*^flox/flox^; Osterix–cyclic recombinase [Osx-cre]), Qi et al.^(82)^ showed a blunted anabolic response. Taken together, the evidence supports a relationship between autophagy and iPTH.

Canonical Wnt signaling promotes osteoblast expansion and function. Soluble ligands bind to the receptors (including LRPs) that induce stabilization of β catenin (β-cat), allowing it to translocate to the nucleus and alter gene expression.^(83)^ In mice with mutations in Lrp6 and β-cat, there were similar anabolic responses to PTH (vertebrae and femur when β-cat deletion was under control of dentin matrix acidic phosphoprotein 1 [DMP1], and in the vertebrae when under control of Osx). Other Wnt family member proteins have been studied with iPTH, and it is clear that this pathway is critical for its anabolic effects in bone. N-cadherin restrains Wnt signaling and bone formation in osteoblasts.^(84)^ Interestingly, when the gene for N-cadherin, *Cdh2*, is disrupted, the anabolic response to iPTH is increased. When both positive and negative regulators of Wnts are affected, the response to iPTH increases, suggesting anabolic PTH is sensitive to slight changes in Wnts.

N-cadherin may affect PTH responsiveness through other mechanisms as well. Expression of *Cdh2* is increased with maturity of osteoblasts and decreased expression is associated with osteosarcoma.^(85,86)^ N-cadherin mediates cell-to-cell adhesion, highlighting the effect of interaction with the microenvironment on osteoblasts. Mdx mice have a mutation in dystrophin, a protein that also helps osteoblasts interact with their environment by connecting the cytoplasm to the extracellular matrix in a complex. Disruption in dystrophin function increases the anabolic response to iPTH. Both N-cadherin and dystrophin are affected by calcium. N-cadherin is a calcium dependent glycoprotein, whereas Mdx mice exhibit increased intracellular calcium levels.^(87)^ It is possible that these changes in calcium regulation alter responsiveness to iPTH.

This work summarizes decades of work aimed to outline the mechanisms of anabolic iPTH, with more studies surely forthcoming. The reports described highlight the importance of many cell types in the bone microenvironment. Signaling starts in the osteoblast, depends on intracellular second messengers, and is then affected by/affects microenvironmental cues and other organ systems, formulating a complex and dynamic process that results in bone formation and bone accrual. The insights from the analysis of the pooled data provide better direction for future experiments and appropriate interpretation.

## Supplementary Material

supplement

## Figures and Tables

**TABLE 1.** Statistical analysis of the trabecular bone response in wild-type mice

	Pearson’s correlation		Linear regression of the slope	
Category	*r* ^2^	*p*	Slope	95% CI
Gender				
Female (*n* = 44)	0.8990	<0.0001	1.031	0.8746 to 1.1870
Male (*n* = 40)	0.7698	<0.0001	1.808	1.3160 to 2.3010
Both (*n* = 11)	0.3470	0.2957	0.748	−0.7763 to 2.2720
Bone site				
Tibia (*n* = 15)	0.8631	<0.0001	1.194	0.9090 to 1.4790
Femur (*n* = 63)	0.5204	<0.0001	1.690	1.2750 to 2.1050
Vertebrae (*n* = 21)	0.1462	0.0872	0.620	−0.0996 to 1.3400
Age at start of treatment				
0–2 weeks (*n* = 12)	0.4261	0.0214	0.988	0.1802 to 1.7970
4–8 weeks (*n* = 22)	0.3150	0.0066	0.752	0.2348 to 1.2690
9–10 weeks (*n* = 23)	0.6942	<0.0001	0.950	0.6640 to 1.2360
11–12 weeks (*n* = 25)	0.7071	<0.0001	1.530	1.1050 to 1.9540
>12 weeks (*n* = 22)	0.6239	<0.0001	2.031	1.2950 to 2.7670
Days per week of treatment				
5–5.5 (*n* = 35)	0.8758	<0.0001	1.250	1.0060 to 1.4940
7 (*n* = 66)	0.6487	<0.0001	1.3178	0.9320 to 1.7010
Treatment duration				
<4 weeks (*n* = 23)	0.6880	<0.0001	1.347	0.9357 to 1.7590
4 weeks (*n* = 48)	0.3858	<0.0001	0.885	0.5335 to 1.2016
5–6 weeks (*n* = 22)	0.6749	<0.0001	2.459	1.6630 to 3.2550
7–12 weeks (*n* = 12)	0.6503	0.0015	0.790	0.3814 to 1.1970
Treatment dose (μg/kg/day)				
≦30 (*n* = 19)	0.6201	<0.0001	2.176	1.3050 to 3.0480
40 (*n* = 19)	0.6799	<0.0001	1.565	1.0150 to 2.1140
50–60 (*n* = 13)	0.3717	0.0269	1.135	0.1559 to 2.1150
80 (*n* = 44)	0.4488	<0.0001	0.919	0.6021 to 1.2370
90–160 (*n* = 10)	0.9454	<0.0001	1.001	0.8050 to 1.1970

*Notes*: Data was pooled to analyze Pearson’s correlation of the trabecular response of wild-type mice to vehicle or iPTH. The *r*^2^ and *p* value are reported from this analysis. The slope and 95% CI of the linear regression of the slope is also reported.

Abbreviations: CI, confidence interval; iPTH, intermittent parathyroid hormone.

**TABLE 2.** Genetic models treated with iPTH

Target gene	Genotype	Gender	PTH regimen	Age of mice during treatment	Bone site	FC in trabecular BV/TV	N.Ob/BS	N.Oc/BS	Strain	Year	Reference
1α(OH) ase	*1α(OH)ase* ^*−/−*^	♂	40 μg/kg/day hPTH(1–34)	12–16 weeks	Tibia	~1.101	No change	No change	C57BL/6J; BALB/c	2008	^(15)^
Ampkα1	*Ampka1* ^*−/−*^	NI	80 μg/kg/day hPTH(1–34) (5 days/week)	12–16 weeks	Tibia	~4.250	ND	ND	C57BL/6129/SV	2012	^(16)^
Areg	*Areg* ^*−/−*^	♀	80 μg/kg/day hPTH(1–34) (5 days/week)	12–16 weeks	Femur	~15.75	ND	Decreased	129/C57BL/6	2015	^(17)^
Atf4	*Amf4* ^*−/−*^	NI	60 μg/kg/day hPTH(1–34)	5–33 days	Femur	~0.468	ND	ND	Swiss Black	2009	^(18)^
Atf4	*Atf4* ^*−/−*^	NI	60 μg/kg/day hPTH(1–34)	5–33 days	Vertebrae	~0.353	ND	ND	Swiss Black	2009	^(18)^
Bcl2	*Bcl2* ^*−/−*^	NI	50 μg/kg/day hPTH(1–34)	4–13 days	Tibia	1.054	ND	ND	129/C57BL/6	2009	^(19)^
Bcl2	*Bcl2*^*−/−*^ *Bim*^*+/−*^	♂	80 μg/kg/day hPTH(1–34)	16–20 weeks	Tibia	ND	ND	No change	C57BL/6 (10th generation)	2010	^(20)^
β-arr2	*β-arr2* ^*−/−*^	♂	80 μg/kg/day hPTH(1–34) (5 days/week)	12–16 weeks	Femur	ND	Increased	Increased	C57Bl/6	2005	^(21)^
β-arr2	*β-arr2* ^*−/−*^	♂	80 μg/kg/day hPTH(1–34) (5 days/week)	12–16 weeks	Vertebrae	~0.000	ND	ND	C57Bl/6	2005	^(21)^
β-arr2	*β-arr2* ^*−/−*^	♂	40 μg/kg/day hPTH(1–34)	9–17 weeks	Vertebrae	~0.428	Decreased	Decreased	C57Bl/6	2009	^(22)^
β-arr2	*β-arr2* ^*−/−*^	♂	40 μg/kg/day hPTH(1–34)	9–17 weeks	Tibia	~0.179	ND	ND	C57Bl/6	2009	^(22)^
β-cat	*Dmp1-CreERt2;β-cat* ^*f/f*^	♂	30 μg/kg/day rhPTH(1–34)	12.5–17.5 weeks	Femur	~2.115	ND	ND	C57Bl/6 129	2016	^(23)^
β-cat	*Dmp1-CreERt2;β-cat* ^*f/f*^	♂	30 μg/kg/day rhPTH(1–34)	12.5–17.5 weeks	Vertebrae	~2.571	ND	ND	C57Bl/6 129	2016	^(23)^
β-cat	*Osx-Cre;β-cat* ^*f/f*^	♂	80 μg/kg/day rhPTH(1–34)	7–11 weeks	Femur	~1.120	ND	ND	C57Bl/6 (6th generation)	2018	^(24)^
β-cat	*Osx-Cre;β-cat* ^*f/f*^	♂	80 μg/kg/day rhPTH(1–34)	7–11 weeks	Vertebrae	~3.350	ND	ND	C57Bl/6 (6th generation)	2018	^(24)^
β_2_AR	*Adbr* ^*−/−*^	♀	80 μg/kg/day hPTH(1–34) (5 days/week)	10–14 weeks	Femur	~ −0.081	ND	Decreased	C57Bl/6	2012	^(25)^
β_2_AR	*Adbr* ^*−/−*^	♀	80 μg/kg/day hPTH(1–34)(5 days/week)	10–14 weeks	Vertebrae	~ −0.131	ND	ND	C57Bl/6	2012	^(25)^
β_2_AR	*Adbr* ^*−/−*^	♀	80 μg/kg/day hPTH(1–34) (5 days/week)	54–58 weeks	Femur	~ −0.113	ND	No change	C57Bl/6	2012	^(25)^
BMI1	*Bmi1* ^*−/−*^	♀♂	80 μg/kg/day hPTH(1–34)	1–4 weeks	Femur	Cannot determine (missing necessary controls)	Cannot determine (missing necessary controls)	ND	129Ola FVB/N hybrid	2014	^(26)^
Bmp2, Bmp4	*R26CreER/R26CreER and Bmp2*^*c/c*^*. Bmp2*^*c/c*^*; Bmp4*^*c/c*^*; R26Cre*^*ER/+*^ *(Bmp2/4 DCKO); OVX*	♀	40 μg/kg/day hPTH(1–34) (5 days/week)	10–12 to 16–18 weeks	Femur	Cannot determine (missing necessary controls)	ND	ND	NI	2016	^(27)^
BSP	*Bsp* ^*−/−*^	♂	0.8 μg/μl PTH 1–84 (local injection)	12–14 weeks	Calvaria	~0.985 (BV reported)	ND	ND	129/CD-1	2015	^(28)^
C-FMS	*MAFIA*	♀	50 μg/kg/day hPTH(1–34)	16–22 weeks	Tibia	~0.127	ND	Decreased	C57Bl/6J	2014	^(29)^
C-FOS	*c-fos* ^*−/−*^	NI	50 μg/kg/day hPTH(1–34)	4–21 days	Vertebrae	~0.316	ND	ND	C57Bl/6 (5th generation)	2002	^(30)^
CaSR	^*Col-Bone*^ *CaSR* ^*Δflox/Δflox*^	NI	50 μg/kg/day hPTH(1–34)	4–17 days	Tibia	~0.893	ND	ND	C57Bl/6 CD-1	2015	^(31)^
CD40L	*CD40L* ^*−/−*^	♀	80 μg/kg/day hPTH(1–34)	12–16 weeks	Femur	0.135	ND	Decreased	C57Bl/6	2014	^(32)^
Cdh2	*Osx-Cre::Cdh2* ^*f/f*^	♂	80 μg/kg/day hPTH(1–34) (5 days/week)	4 weeks of iPTH starting 12–16 weeks	Tibia	3.815	No change	Decreased	C57Bl/6	2014	^(33)^
Cdh2	*Dmp1-cre;Cdh2* ^*f/f*^	♂	80 μg/kg/day hPTH(1–34) (5 days/week)	8–12 weeks	Femur	3.393	Increased	Increased	C57Bl/6	2016	^(34)^
Cdk1	*Osx-Cre;Cdk1* ^*f/f*^	♀	80 μg/kg/day hPTH(1–34) (5 days/week)	12–16 weeks	Vertebrae	~2.018	Increased	No change	C57Bl/6129S6/SvEvTac	2018	^(35)^
Cox2	*COX2* ^*−/−*^	♂	80 μg/kg/day hPTH(1–34)	20–23 weeks	Femur	1.669	Increased	No change	CD-1 (9th generation)	2010	^(36)^
Cox2	*COX2* ^*−/−*^	♂	80 μg/kg/day hPTH(1–34)	20–23 weeks	Vertebrae	5.688	ND	ND	CD-1 (9th generation)	2010	^(36)^
Crem	*CrerrT* ^*−/−*^	♂	160 μg/kg/day hPTH(1–34)	10 days of iPTH from 11–12 weeks	Femur	~0.312	No change	Increased	129Sv; C57Bl/6	2007	^(37)^
Cx43	*Cx43* ^*ΔCT/fl*^ *;DMP1–8kb-Cre*	♀	100 μg/kg/day hPTH(1–34)	16–18 weeks	Femur	1.154	ND	ND	C57Bl/6	2015	^(38)^
Dkk1	*Dkk1 TG; 2.3-kb rat collagen type Ia promoter*	NI	95 μg/kg/day hPTH(1–34)	34 days of iPTH from 12–14 weeks	Tibia	ND	Decreased	Decreased	C57Bl/6 CD-1	2011	^(39)^
Egdr	*Egfr*^*Wa5*^ *(impaired EGFR signaling)*	♀	80 μg/kg/day hPTH(1–34) (5 days/week)	12–16 weeks	Femur	~0.704**	ND	Decreased	C57Bl/6	2012	^(40)^
Fgf2	*Fgf2* ^*−/−*^	♂	80 μg/kg/day hPTH(1–34)	8–12 weeks	Femur	0.647	Decreased	No change	Black Swiss 129Sv	2006	^(41)^
Fgf2	*Fgf2* ^*−/−*^	♀	80 μg/kg/day hPTH(1–34)	60–64 weeks	Femur	0.139	ND	ND	Black Swiss 129Sv	2006	^(41)^
Fgf2	*3.6Col1GFPsaph* ^*tg/tg*^ *; Fgf2* ^*−/−*^	♀	20 μg/kg/day PTH(1–34)	12 weeks (8 h)	Tibia	ND	ND	ND	Black Swiss 129Sv; FVB/N	2018	^(42)^
Fgf23	*Fgf23* ^*−/−*^	NI	100 μg/kg/day hPTH(1–34)	8–22 days	Femur	~1.077	No change	ND	C57Bl/6 129Sv	2011	^(43)^
Fgfr3	*Fgfr3* ^*−/−*^	♂	80 μg/kg/day hPTH(1–34)	16–20 weeks	Femur	~2.533	Decreased	Increased	C3H	2016	^(44)^
Fgfr3	*FGFR3* ^*G369C/+*^	NI	80 μg/kg/day hPTH(1–34)	8–12 weeks	Femur	~2.814	ND	ND	C57Bl/6	2017	^(45)^
Ghr	*DMP1-Cre;GHR* ^*f/f*^	♀	80 μg/kg/day hPTH(1–34)	4–8 weeks	Femur	0.234	Decreased	No change	C57Bl/6	2015	^(46)^
GPR126	*Osx-cre;Gprl 26* ^*f/f*^	♀♂	80 μg/kg/day hPTH(1–34)	5–30 days	Femur	~1.975	ND	ND	C57Bl/6	2020	^(47)^
GRK2	*GRK1* ^*TG*^ *;1.3kb fragment of OG2 promoter*	♀♂	40 μg/kg/day hPTH(1–34)	36–40 weeks	Vertebrae	Cannot determine (missing necessary controls)	Increased	No change	B6SJLF1/J	2009	^(48)^
Gα_s_	*Gα*_*s*_^*Osx-KO*^	♀♂	80 μg/kg/day hPTH(1–34) (5 days/week)	8–12 weeks	Femur	0.223	Increased	Increased	C57Bl/6 CD1	2016	^(49)^
HDAC4	*HDAC4* ^*fl/fl*^ *; DMP1-cre*	♀	100 μg/kg/day hPTH(1–34) (5 days/week)	8–12 weeks	NI	~0.971	ND	ND	C57Bl/6	2016	^(50)^
HDAC4; HDAC5	*HDAC5T* ^*−/−*^ *; HDAC* ^*fl/fl*^ *; DMP1-cre*	♀	100 μg/kg/day hPTH(1–34) (5 days/week)	8–12 weeks	NI	~2.111	ND	ND	C57Bl/6	2016	^(50)^
HDAC5	*HDAC5* ^*−/−*^	♀	100 μg/kg/day hPTH(1–34) (5 days/week)	8–12 weeks	NI	~2.111	ND	ND	C57Bl/6	2016	^(50)^
Hif-1α	*Ocn-Cre;Hif1αf/f*	♀	20 μg/kg/day hPTH(1–34)	10–16 weeks	Femur	~1.511	ND	ND	C57Bl/6	2014	^(51)^
Hif-1α	*Ocn-Cre;Hif1αf/f*	♀	40 μg/kg/day hPTH(1–34)	10–16 weeks	Femur	~1.223	ND	ND	C57Bl/6	2014	^(51)^
Igf-1	*Igf-1* ^*−/−*^	NI	160 μg/kg/day hPTH(1–34)	5–6.5 weeks	Femur	ND	ND	ND	NI	2001	^(52)^
Igf-1	*B6.C3H-6T*	♀	50 μg/kg/day hPTH(1–34)	16–20 weeks	Femur	0.704	ND	ND	C57Bl/6 (10th generation)	2005	^(53)^
Igf-1	*Igf1* ^*fl/fl*^ *; Albumin-Cre*	♂	50 μg/kg/day hPTH(1–34) (5 days/week)	12–16 weeks	Vertebrae	~2.150	ND	ND	FVB/N, C57BL, and 129Sv	2006	^(54)^
Igf-1	*ALS* ^*−/−*^	♂	50 μg/kg/day hPTH(1–34) (5 days/week)	12–16 weeks	Vertebrae	~ −0.300	ND	ND	C57Bl/6 (6th generation)	2006	^(54)^
Igf-1	*Igf1* ^*fl/fl*^ *; Albumin-Cre; ALS* ^*−/−*^	♂	50 μg/kg/day hPTH(1–34) (5 days/week)	12–16 weeks	Vertebrae	~ −0.350	ND	ND	FVB/N; C57BL 129Sv	2006	^(54)^
Igf-1	*HIT (hepatic IGF-1 transgene)*	♂	50 μg/kg/day hPTH(1–34)	12–16 weeks	Femur	~1.622	ND	ND	FVB/N	2010	^(55)^
Igf-1	*HIT KO*	♂	50 μg/kg/day hPTH(1–34)	12–16 weeks	Femur	~2.069	ND	ND	FVB/N	2010	^(55)^
IGF-IR	*Ocn-Cre;Igf-IR* ^*f/f*^	*NI*	80 μg/kg/day rat PTH(1–34)	12–14 weeks	Tibia and femur	ND	ND	ND	FVB/N	2014	^(56)^
IL18	*IL18* ^*−/−*^	♀	80 μg/kg/day hPTH(1–34) (5 days/week)	4 weeks of iPTH starting at 7–8 weeks	Tibia and femur	ND	ND	ND	DBA/1	2008	^(57)^
IL6	*lL6* ^*−/−*^	♀♂	50 μg/kg/day hPTH(1–34)	3–24 days	Femur	~0.596	ND	Decreased	C57Bl/6	2013	^(58)^
IL6	*IL6* ^*−/−*^	♀♂	50 μg/kg/day hPTH(1–34)	16–22 weeks	Femur	~3.333	ND	ND	C57Bl/6	2013	^(58)^
lrs-1	*lrs-1* ^*−/−*^	♂	80 μg/kg/day hPTH(1–34)	10–14 weeks	Tibia and femur	0.090	No change	Decreased	C57Bl6 CBA	2005	^(59)^
lrs-2	*lrs-2* ^*−/−*^	♂	80 μg/kg/day hPTH(1–34)	10–14 weeks	Tibia and femur	2.499	Decreased	Decreased	C57Bl6 CBA	2005	^(59)^
Kl	*Kl* ^*−/−*^	NI	100 μg/kg/day hPTH(1–34)	8–22 days	Femur	~1.077	No change	ND	C57Bl/6 129Sv	2010	^(43)^
Ldlr	*Ldlr* ^*−/−*^	♀	40 μg/kg/day hPTH(1–34) (5 days/week)	20–25 weeks	Femur	0.624	Increased	Increased	C57Bl/6	2009	^(60)^
Ldlr	*Ldlr* ^*−/−*^ *; pOBCol3.6GFPtpz and pOBCol2.3GFPCyan*	♀	40 μg/kg/day hPTH(1–34) (5 days/week)	5 weeks of iPTH starting at 8–12 weeks	Calvaria	ND	Decreased	ND	C57Bl/6	2013	^(61)^
Ldlr	*Ldlr* ^*−/−*^ *; pOBCol3.6GFPtpz and pOBCol2.3GFPCyan*	♀	40 μg/kg/day hPTH(1–34) (5 days/week)	5 weeks of iPTH starting at 8–12 weeks	Femur	ND	Decreased	ND	C57Bl/6	2013	^(61)^
Lrp5	*Lrp5* ^*−/−*^	♀♂	40 μg/kg/day hPTH(1–34) (5 days/week)	12–16 weeks	Hindlimb	ND	ND	ND	129S/J	2006	^(62)^
Lrp5	*Lrp5* ^*−/−*^	♀	80 μg/kg/day hPTH(1–34) (every other day)	20–26 weeks	Femur	~0.435	ND	ND	C57Bl/6	2009	^(63)^
Lrp5	*Lrp5* ^*−/−*^	♂	80 μg/kg/day hPTH(1–34) (every other day)	20–26 weeks	Femur	~ −1.294	ND	ND	C57Bl/6	2009	^(63)^
Lrp5	*Lrp5* ^*−/−*^	♀	80 μg/kg/day hPTH(1–34) (every other day)	20–26 weeks	Vertebrae	~10.000	No change	No change	C57Bl/6	2009	^(63)^
Lrp5	*Lrp5* ^*−/−*^	♂	80 μg/kg/day hPTH(1–34) (every other day)	20–26 weeks	Vertebrae	~ −1.028	No change	No change	C57Bl/6	2009	^(63)^
Lrp6	*Ocn-cre;Lrp6* ^*f/f*^	♂	80 μg/kg/day hPTH(1–34) (5 days/week)	8–12 weeks	Femur	~ −1.255	Decreased	No change	C57Bl/6J; 129 FVB/N	2013	^(64)^
Lrp6	*Ocn-Cre;Lrp6* ^*f/f*^	♂	80 μg/kg/day hPTH(1–34) (5 days/week)	8–12 weeks	Femur	ND	ND	ND	C57Bl/6J; 129 FVB/N	2015	^(65)^
MCP-1	*Mcp-1* ^*−/−*^	♂	80 μg/kg/day hPTH(1–34) (5 days/week)	16–22 weeks	Tibia	~0.084	ND	Decreased	C57Bl/6	2013	^(66)^
MCP-1	*Mcp-1* ^*−/−*^	♀♂	80 μg/kg/day hPTH(1–34) (5 days/week)	20–26 weeks	ND	ND	ND	ND	C57Bl/6	2013	^(66)^
Mdx	*C57BL/10ScSn/DMD-mdx*	♂	30 μg/kg/day black bear PTH (1–61,63,64, 67–87) (5 days/week)	4–10 weeks	Femur	~5.833	No change	Decreased	C57BL/610ScSn	2012	^(88)^
Mfge8	*Mfge8* ^*−/−*^	♀♂	50 μg/kg/day hPTH(1–34)	16–22 weeks	Tibia	~2.000 (reported as FC)	ND	Decreased	C57Bl/6	2018	^(89)^
MHC I	*MHC I* ^*−/−*^	NI	80 μg/kg/day hPTH(1–34)	5–9 weeks	Femur	~0.173	ND	ND	C57Bl/6	2009	^(72)^
MHC I; MHC II	*MHC I* ^*−/−*^ *; MHC II* ^*−/−*^	NI	80 μg/kg/day hPTH(1–34)	5–9 weeks	Femur	~0.058	ND	ND	C57Bl/6	2009	^(72)^
MHC II	*MHC II* ^*−/−*^	NI	80 μg/kg/day hPTH(1–34)	5–9 weeks	Femur	~1.038	ND	ND	C57Bl/6	2009	^(72)^
miR-29-3p	*miR-29-3p decoy*	♀	80 μg/kg/day hPTH(1–34) (5 days/week)	12–16 weeks	Femur	~8.858	Increased	No change	C57Bl/6	2020	^(90)^
Mkp1	*Mkp1* ^*−/−*^	♀	50 μg/kg/day hPTH(1–34) (5–6 days/week)	3–24 days	Femur	~1.250 (reported as FC)	ND	ND	C57Bl/6 129	2011	^(91)^
Nf1	*Nf1* ^*+/−*^	♂	80 μg/kg/day hPTH(1–34)	28 days of iPTH starting 8–12 weeks	Tibia	~0.963	ND	Increased	C57Bl/6	2006	^(92)^
Nmp4	*Nmp4* ^*−/−*^	♀	30 μg/kg/day hPTH(1–34)	10–17 weeks	Femur	~2.906	ND	ND	C57Bl/6 (6th generation)	2009	^(93)^
Nmp4	*Nmp4* ^*−/−*^	♀	30 μg/kg/day hPTH(1–34)	10–12 weeks	Tibia	~1.500	ND	ND	C57Bl/6 (6th generation)	2011	^(94)^
Nmp4	*Nmp4* ^*−/−*^	♀	30 μg/kg/day hPTH(1–34)	10–17 weeks	Tibia	~0.800	ND	ND	C57Bl/6 (6th generation)	2011	^(94)^
Nmp4	*Nmp4* ^*−/−*^	♀	30 μg/kg/day hPTH(1–34)	10–12 weeks	Vertebrae	~1.467	ND	ND	C57Bl/6 (6th generation)	2011	^(94)^
Nmp4	*Nmp4* ^*−/−*^	♀	30 μg/kg/day hPTH(1–34)	10–17 weeks	Vertebrae	~4.206	ND	ND	C57Bl/6 (6th generation)	2011	^(94)^
Nmp4	*Nmp4* ^*−/−*^	♀	30 μg/kg/day hPTH(1–34)	10–13 weeks	Femur	~2.523	ND	ND	C57Bl/6 (6th-7th generation)	2012	^(95)^
Ocn	*Ocn* ^*−/−*^	♀	80 μg/kg/day hPTH(1–34) (5 days/week)	10–14 weeks	Vertebrae	1.266	ND	ND	C57Bl/6	2008	^(96)^
Ocn	*Ocn* ^*−/−*^	♀	80 μg/kg/day hPTH(1–34) (5 days/week)	10–14 weeks	Femur	1.174	No change	Increased	C57Bl/6	2008	^(96)^
Opn	*Opn* ^*−/−*^	♀	80 μg/kg/day hPTH(1–34) (5 days/week)	7–11 weeks	Tibia and femur	~1.362**	ND	Decreased	129	2003	^(97)^
OSMR	*Osmr* ^*−/−*^	♂	30 μg/kg/day hPTH(1–34) (5 days/week)	6–9 weeks	Tibia	~ −0.518	Decreased	Increased	C57Bl/6	2011	^(98)^
p38α	*Ocn-Cre;p38α* ^*f/f*^	♂	40 μg/kg/day hPTH(1–34)	12–16 weeks	Femur	~0.415	Decreased	Decreased	C57Bl/6	2015	^(99)^
Pappa	*Pappa* ^*−/−*^	♀	80 μg/kg/day hPTH(1–34) (5 days/week)	12–18 weeks	Femur	~0.277**	ND	ND	C57Bl/6; 129	2015	^(100)^
PLS3	*Pls3* ^*−/0*^	♂	80 μg/kg/day hPTH(1–34)	10–12 weeks	Vertebrae	ND	No change	ND	C57Bl/6	2020	^(101)^
Postn	*Postn−/−*	♀	40 μg/kg/day hPTH(1–34)	12–17 weeks	Femur	1.106	ND	Increased	C57Bl/6	2012	^(102)^
Postn	*Postn* ^*−/−*^	♀	40 μg/kg/day hPTH(1–34)	12–17 weeks	Vertebrae	1.762	ND	ND	C57Bl/6	2012	^(102)^
Prg4	*Prg4* ^*−/−*^	♀♂	50 μg/kg/day hPTH(1–34)	4–21 days	Femur	1.239	ND	ND	C57Bl/6	2012	^(71)^
Prg4	*Prg4* ^*−/−*^	♀♂	50 μg/kg/day hPTH(1–34)	16–22 weeks	Femur	−9.692	No change	Decreased	C57Bl/6	2012	^(71)^
PTH and 1α(OH) ase	*PTH* ^*−/−*^ *;1α(OH)ase* ^*−/−*^	NI	0.2 μg/kg/day rat PTH(1–34)/day	4–14 days	Femur	~62.000	Cannot determine (no reported WT+PTH)	Cannot determine (no reported WT+PTH)	C57Bl/6J and BALB/c	2005	^(103)^
PTH1R	*Lck-Cre;PTH1R* ^*f/f*^	♀	80 μg/kg/day hPTH(1–34)	2–6 weeks	Femur	0.409	Decreased	No change	C57Bl/6	2012	^(104)^
PTH1R	*Lck-Cre;PTH1R* ^*f/f*^	♀	80 μg/kg/day hPTH(1–34)	13–17 weeks	Femur	−0.314	ND	ND	C57Bl/6	2012	^(104)^
PTH1R	*pdPTH1R*	♀	40 μg/kg/day hPTH(1–34) (5 days/week)	12–22 weeks	Vertebrae	~0.837	ND	ND	C57Bl/6	2012	^(105)^
PTH1R	*pdPTH1R*	♂	40 μg/kg/day hPTH(1–34) (5 days/week)	12–22 weeks	Vertebrae	~0.890	ND	ND	C57Bl/6	2012	^(105)^
PTH1R	*pdPTH1R*	♀	40 μg/kg/day hPTH(1–34) (5 days/week)	12–22 weeks	Femur	~0.822	ND	ND	C57Bl/6	2012	^(105)^
PTH1R	*pdPTH1R*	♂	40 μg/kg/day hPTH(1–34) (5 days/week)	12–22 weeks	Femur	~1.000	ND	ND	C57Bl/6	2012	^(105)^
PTH1R	*DMP1-Cre;PTH1R* ^*f/f*^	♀	80 μg/kg/day hPTH(1–34) (5 days/week)	4 weeks of iPTH (start age NI)	Femur	ND	ND	ND	C57Bl/6 dominant (mixed background)	2013	^(106)^
PTH1R	*DMP1-Cre;PTH1R* ^*f/f*^	♀	80 μg/kg/day hPTH(1–34) (5 days/week)	4 weeks of iPTH (start age NI)	Vertebrae	0.339	ND	ND	C57Bl/6 dominant (mixed background)	2013	^(106)^
PTH1R	*DMP1-Cre;PTH1R* ^*f/f*^	♀	100 ng/g/day PTH(1–34)	16–20 weeks	Femur	~0.739	ND	ND	C57BL/6Nhsd	2016	^(107)^
PTH1R	*DMP1-Cre;PTH1R* ^*f/f*^	♂	100 ng/g/day PTH(1–34)	16–20 weeks	Femur	~ −0.081	ND	ND	C57BL/6Nhsd	2016	^(107)^
PTHRP	*Pthrp* ^*+/−*^	♂	40 μg/kg/day hPTH(1–34)	12–24 weeks	Femur	~10.230	ND	ND	FVB/N CD-1	2005	^(14)^
Rac1	*Osx-Cre;Rac1* ^*−/−*^	NI	80 μg/kg/day hPTH(1–34)	4–8 weeks	Femur	ND	NI	NI	NI	2020	^(108)^
Rac2	*Rac2* ^*−/−*^	NI	80 μg/kg/day hPTH(1–34)	12–16 weeks	Tibia	ND	Increased	Increased	C57Bl/6 (used as control)	2008	^(109)^
Rag2	*Rag2* ^*−/−*^	NI	80 μg/kg/day hPTH(1–34)	5–9 weeks	Femur	~0.406	ND	ND	C57Bl6/J	2009	^(72)^
RAGE	*RAGE* ^*−/−*^	♀	30 μg/kg/day hPTH(1–34)	10–12 weeks	Femur	~0.00	ND	ND	C57Bl/6	2010	^(110)^
RAGE	*RAGE* ^*−/−*^	♀	30 μg/kg/day hPTH(1–34)	10–17 weeks	Femur	~0.495	ND	ND	C57Bl/6	2010	^(110)^
RAGE	*RAGE* ^*−/−*^	♀	30 μg/kg/day hPTH(1–34)	10–12 weeks	Vertebrae	~1.857	ND	ND	C57Bl/6	2010	^(110)^
Runx2	*Runx2 Tg*	♀	100 μg/kg/day hPTH(1–34)	4–10 weeks	Femur	~0.637	ND	Increased	C57Bl/6	2007	^(111)^
sFRP1	*sFRP* ^*−/−*^	♀	100 μg/kg/day hPTH(1–34)	8–12 weeks	Femur	~0.711 (reported as FC)	ND	ND	C57Bl/6 (albino)-129SvEv (LEX-1)	2006	^(112)^
sFRP1	*sFRP* ^*−/−*^	♀	100 μg/kg/day hPTH(1–34)	24–28 weeks	Femur	~0.627 (reported as FC)	ND	ND	C57Bl/6 (albino)-129SvEv (LEX-1)	2006	^(112)^
sFRP1	*sFRP* ^*−/−*^	♀	100 μg/kg/day hPTH(1–34)	36–40 weeks	Femur	~0.332 (reported as FC)	ND	ND	C57Bl/6 (albino)-129SvEv (LEX-1)	2006	^(112)^
sFRP1	*sFRP1 Tg*	♀	40 μg/kg/day hPTH(1–34) (5 days/week)	12–14 weeks	Femur	0.103	ND	No change	FVB/N-Swiss Webster hybrid	2010	^(113)^
sFRP1	*sFRP1 Tg*	♂	40 μg/kg/day hPTH(1–34) (5 days/week)	12–14 weeks	Femur	0.120	ND	No change	FVB/N-Swiss Webster hybrid	2010	^(113)^
sFRP1	*sFRP1 Tg*	♀	40 μg/kg/day hPTH(1–34) (5 days/week)	12–14 weeks	Vertebrae	0.099	ND	ND	FVB/N-Swiss Webster hybrid	2010	^(113)^
sFRP1	*sFRP1 Tg*	♂	40 μg/kg/day hPTH(1–34) (5 days/week)	12–14 weeks	Vertebrae	0.402	ND	ND	FVB/N-Swiss Webster hybrid	2010	^(113)^
Sost	*Sost TG*	♂	100 μg/kg/day hPTH(1–34) (5–6 days/week)	24–33 weeks	Femur	0.391	ND	No change	FVB, C57Bl/6	2010	^(114)^
Sost	*Sost* ^*−/−*^	♂	30 μg/kg/day hPTH(1–34)	10–16 weeks	Femur	~0.779	ND	ND	129/SvJ and Black Swiss	2011	^(115)^
Sost	*Sost* ^*−/−*^	♂	90 μg/kg/day hPTH(1–34)	10–16 weeks	Femur	~0.877	ND	ND	129/SvJ and Black Swiss	2011	^(115)^
TCRβ	*TCRβ* ^*−/−*^	NI	80 μg/kg/day hPTH(1–34)	5–9 weeks	Femur	0.503	Decreased	Increased	C57Bl/6	2009	^(72)^
TGFβ1	*TGFβ1* ^*−/−*^ *,Rag2* ^*−/−*^	♂	40 μg/kg/day hPTH(1–34) (5 days/week)	8–12 weeks	Tibia	~ −0.388	Decreased	No change	C57Bl/6	2011	^(116)^
TGIF1	*Tgif1* ^*fl/fl*^ *; DMP1-cre*	♂	100 μg/kg/day hPTH(1–34) (5 days/week)	8–12 weeks	Tibia	~0.103	Decreased	No change	C57Bl/6	2019	^(117)^
TGIF1	*Tgif1* ^*−/−*^	♂	100 μg/kg/day hPTH(1–34) (5 days/week)	8–12 weeks	Tibia	~ −0.126	Decreased	Decreased	C57Bl/6	2019	^(117)^
Timp1	*Timp1 TG by type-I collagen promoter*	♀	40 μg/kg/day hPTH(1–34)	10–16 weeks	Femur	1.964	ND	Decreased	C57Bl/6 CBA	2006	^(118)^
Ts65Dn	*Mosel for trisomy 21*	♂	30 μg/kg/day hPTH(1–34)	12–16 weeks	Tibia	~1.450	No change	No change	C57Bl/6; C3H/HeJ	2012	^(119)^
Ts65Dn	*Mosel for trisomy 21*	♂	80 μg/kg/day hPTH(1–34)	12–16 weeks	Tibia	~1.450	No change	No change	C57Bl/6; C3H/HeJ	2012	^(119)^
Vps35	*Ocn-Cre;Vps35* ^*f/f*^	♂	50 μg/kg/day hPTH(1–34) (5 days/week)	7–12 weeks	Femur	~7.690	ND	ND	C57Bl/6	2016	^(120)^
Wnt1	*Wnt1* ^*+/R235W*^	♀	80 μg/kg/day hPTH(1–34)	52–56 weeks	Femur	ND	ND	ND	C57Bl/6 129	2020	^(121)^

*Notes*: A summary of each publication using iPTH in a genetic model is alphabetized by target gene. The genotype, gender, PTH regimen, age of mice during treatment, bone site, fold change in BV/TV comparing targeted gene versus WT (target gene/WT), N.Ob/BS, N.Oc/BS, strain, and year are listed.

Abbreviations: ♂, male; ♀, female; ~, values estimated from a graph; **, bone area reported; BV/TV, trabecular bone volume per total volume; FC, fold change; hPTH, human parathyroid hormone; iPTH, intermittent parathyroid hormone; ND, not determined; NI, not indicated; N.Ob/BS, number of osteoblasts per bone surface; N.Oc/BS, number of osteoclasts per bone surface; PTH, parathyroid hormone; WT, wild type.

## Data Availability

The data that support the findings of this study are available from the corresponding author upon reasonable request.
