# Correction to: Anabolic actions of PTH in murine models: two decades of insights

**DOI:** 10.1093/jbmr/zjaf167

**Published:** 2026-01-14

Correction Notice:

This is a correction to: Laura E. Zweifler, Amy J. Koh, Stephanie Daignault-Newton, Laurie K. McCauley, 08 June 2021 Journal of Bone and Mineral Research, Volume 36, Issue 10, 1 October 2021, Pages 1979–1998, https://doi.org/10.1002/jbmr.4389.

The published version of this manuscript contains the following errors:

On page 1980 in the first paragraph of Materials and Methods, the 10th line reads: “...[(PTH-Vehicle)/PTH].” The correction should read “...[(PTH-Vehicle)/Vehicle].” While this formula was quoted in error in the text, the corrected formula presented above [(PTH-Vehicle)/Vehicle] was used for all calculations throughout the paper. This typo does not change the essence of the paper. The authors apologize that this typo was not noticed and potentially misled readers who might have implemented this formula when performing their own analyses.

In Figure 2D, the regression plots are correct; except for the fact that the y-axis scale excluded one data point for the >12wks group (blue line). The following graph presented here now corrects the y-axis scale and shows that one missing data point:


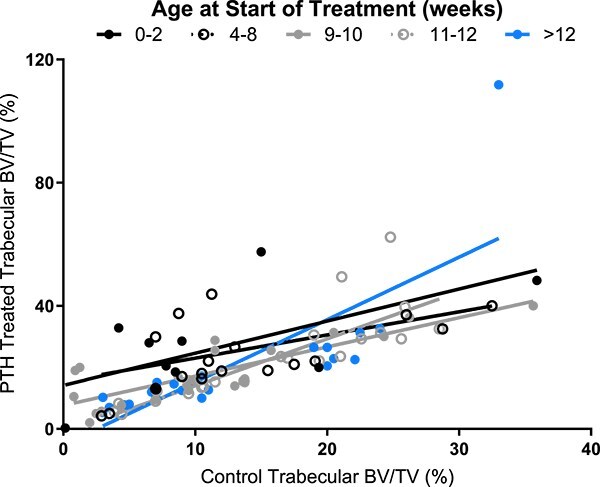


Revised from:


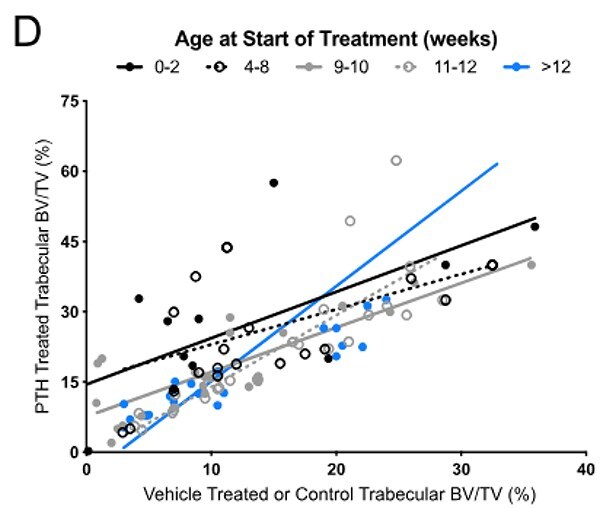


These details have been corrected only in this correction notice to preserve the published version of record.

