# Supplementary material for: Anabolic actions of PTH in murine models: two decades of insights
Source: J Bone Miner Res. Author manuscript; Available in PMC 2021 Dec 14. (PMC8596798; doi:10.1002/jbmr.4389)
Supplement: supplement [file NIHMS1754242-supplement-supplement.docx]

**Anabolic actions of PTH in murine models: Two decades of insights**

**Supplemental Material**

**Actions of iPTH in wildtype mice**

Correlation of the trabecular bone in control- and iPTH-treated wildtype mice were analyzed based on pre-determined categories. For example, males and females (Fig. 2A) respond significantly differently from each other, with males having a greater bone volume response when the control bone volume was above ~12%. Femurs and tibiae (Fig. 2B) respond similarly, with overlapping 95% confidence intervals of the linear regression of the slope. Treatment with iPTH for 5-5.5 days vs. 7 days resulted in insignificant differences (Fig. 2C). The response based on age at the start of treatment (Fig. 2D) suggests that starting at 11-12 weeks or >12 weeks yielded different results than 4-8 weeks or 9-10 weeks, but was only higher if the control bone volume was greater than ~15% for 11-12 weeks or ~19% for >12 weeks. Starting between 4-8 weeks or 9-10 weeks yielded a similar response. For the greatest response, these findings suggest iPTH should be given for 5-6 weeks if control trabecular bone is greater than about 12% (Fig. 2E). This was significantly different from all other intervals (<4 weeks, 4 weeks, and 7-12 weeks). Interestingly, doses of iPTH (Fig. 2F), ≦30, 40, or 50-60 μg/kg/day, were not different in their response. However, 80 μg/kg/day resulted in a significantly smaller response to iPTH than ≦30 or 40 μg/kg/day when the control was higher than ~10% trabecular bone volume. The dose of PTH seems to be inversely related to the response. As stated, this analysis is intended to organize more efficient studies.

**Actions of iPTH anabolic actions in bone using gene targeted mice**

***Receptor Activation and Signaling Pathways***

PTH and PTHrP share the G protein coupled receptor, PTH1R (PTH/PTHrP Receptor, also referred to as PPR). PTHrP haploinsufficient mice (*Pthrp^+/-^*) treated with iPTH exhibit a significant increase in bone volume relative to wildtype controls (10.230-fold increase).^(1)^ This difference is attributed to a possible increase in receptor availability when iPTH is administered. PTH1R activates several G protein-linked systems by coupling to Gα_q/11_ to stimulate phospholipase C, Gα_s_ to stimulate adenylate cyclase, and protein kinase C (PKC) or Gα_12/13_ to simulate phospholipase D. *Pth1r* has been conditionally deleted from osteocytes, using two different promoters for DMP1 (8kb and 10kb). In both studies, authors reported a decreased anabolic response to iPTH.^(2,3)^ Saini et al. suggest that the blunted anabolic response of these mice is due to the control of osteoclasts and osteoblasts via sclerostin (SOST) and receptor activator of nuclear factor kappa-β ligand (RANKL). Later, Deldago-Calle et al. reported that *Sost* mRNA was decreased in loaded ulna of wildtype mice, but unchanged in *PTH1R^fl/fl^-DMP1^cre^* mice. They further explored the effect of bone anabolism in this pathway using a *Sost* transgenic (TG) mouse treated with iPTH and reported that the effect was independent of *Sost*. They concluded that while PTH1R is required in cells expressing *DMP1* (8kb), the anabolic response does not require SOST.

The Gα_s_ subunit has also been under investigation with iPTH treatment.^(4)^ Binding of the PTH1R activates G_s_-dependent pathways ^(5)^, therefore Sinha et al. conditionally knocked out *Gα_s_* from *osterix*-expressing cells, targeting osteoblasts from early in their lineage.^(4)^ Following iPTH treatment, *Gα_s_^Osx-KO^* mice had a reduced anabolic response. Trabecular bone volume in wildtype mice increased with PTH treatment as expected, but *Gα_s_^Osx-KO^* mice had no change in bone volume. Further micro-computed tomography (μCT) analysis showed reduced trabecular number, similar thickness, and increased spacing in the PTH-treated *Gα_s_^Osx-KO^* mice. In response to PTH, *Gα_s_^Osx-KO^* mice had a decreased bone formation rate and increased amount of bone surface covered in osteoblasts and osteoclasts. This demonstrates that the Gα_s_ subunit is downstream of PTH signaling, and highlights its necessity for bone to respond to iPTH.

Activation of the receptor by PTH, as well as its agonists, leads to internalization of the ligand-receptor complex and desensitization of the response to PTH.^(6,7)^ Using a phosphorylation deficient (PD) knock-in mouse model, Datta et al. reported that the PTH receptor does not need to be internalized for PTH anabolic actions.^(8)^ These PD mice had a comparable anabolic response in the vertebrae and femurs as wildtype mice.

G protein coupled receptors are phosphorylated by receptor kinases (GRKs) that can desensitize receptor signaling. GRK activity in osteoblasts enhances activity in those cells, as well as osteoclasts. By overexpressing the C terminus of GRK2 using an osteocalcin gene 2 promoter, GRK activity was attenuated in osteoblasts (GRK2 is not enzymatically active, therefore overexpression can stop G-protein kinase phosphatase activity, and enhance G protein coupled receptors).^(9)^ Using this strategy, the effect of inhibiting GRK activity on the anabolic PTH response was determined.^(10)^ The lumbar spine had increased bone volume in response to iPTH compared to control mice, indicating that when the PTH1R is less susceptible to desensitization, the osteoanabolic effect of iPTH increases. Of note, there were reduced bone forming effects of iPTH in the femoral shaft. Authors postulate that the GRK inhibitor enhances site-specific actions of iPTH, accentuating differences between bone sites.

There is also a subgroup of G protein coupled receptors, termed adhesion G protein coupled receptors, that are defined by large N-terminal extracellular domains and are thought to communicate with adhesive factors.^(11)^ One member, GPR126, is implicated in multiple human skeletal defects, including reduced human height.^(12)^ This phenotype is recapitulated in *Osx-cre; Gpr126^f/f^* mice, and these mice have a delay in osteoblast differentiation and mineralization.^(13)^ However, iPTH treatment rescues the body length phenotype in *Osx-cre; Gpr126^f/f^* mice. Analysis of the femur treated with iPTH shows that these mice have a 1.975-fold increase in trabecular bone volume relative to the response in wildtype controls. This data indicates that PTH might be used to treat shortened stature, and that *Gpr126* plays a role in the anabolic response.

Vacuolar protein sorting 35 (VPS35) is an intracellular protein expressed in osteoblasts and osteoclasts and a component of the cargo-recognition subcomplex of retromer.^(14)^ The retromer complex is essential for recycling transmembrane proteins from the endosome to the golgi apparatus, including PTH1R and the β2-adrenergic receptor (β2AR). Loss of function mutations in *Vps35* impaired translocation of the PTH1R and enhanced PTH signaling while reducing the catabolic PTH response. *Vps35*-deficient mice had lower bone mass, with reduced bone formation and increased resorption.^(15)^ *Vps35^fl/fl^* mice were crossed with *Ocn-Cre* mice to generate a model for targeted *Vps35* deletion in osteoblasts.^(16)^ The anabolic response of iPTH was enhanced in the conditional knockout mice as indicated by a 7.690-fold increase in the relative response of bone volume to iPTH. Xiong et al. reported an interaction with VPS35 and PTH1R trafficking to the Golgi apparatus, leading to a proposed model in which VPS35 turns off PTH1R signaling by interacting with protein phosphatase 1 regulatory subunit 14C (PPP1R14C), reducing the complex with PTH1R. This would allow PP1 phosphatase to dephosphorylate downstream signaling pathways promoting catabolic functions. After treating bone marrow stromal cells from *Vps35^Ocn-Cre^* mice *in vitro* with PTH, there was a similar response to vehicle treatment in the number of osteoclasts (identified as multinucleated TRAP-positive cells). However, after 6 and 12 hours of treatment with PTH (1-34), *Vps35^Ocn-Cre^* bone marrow stromal cells had significantly increased osteocalcin levels, reflecting osteoblastic bone formation activity. This data supports the *in vivo* μCT data, and the interactions between PTH1R and VPS35.

Downstream of PKA phosphorylation is activation of cAMP response element binding protein (CREB), cAMP response element modulator (CREM), and activating transcription factors (ATFs), which are members of the basic leucine zipper transcription factor family that bind to the regulatory regions of genes controlling cAMP response elements (CREs).^(17)^ PTH induces the expression of inducible cAMP early repressor (*Icer*) through the cAMP-PKA pathway. Four ICER products are directed by the P2 promoter region of CREM. The ICER products, in turn, bind to CREs as transcriptional repressors by blocking their access to activators. CREM deficiency reduced the anabolic effect of PTH in both cortical and trabecular compartments.^(18)^ While *Crem*-deficient mice did not have altered bone formation in response to iPTH, osteoclast numbers were increased, which could account for the decreased relative bone volume. This study suggests that *Crem* plays a role in the anabolic response of PTH by limiting osteoclastogenesis.

Recently, transforming growth-interacting factor-1 (*Tgif-1*) was identified as a regulator of bone remodeling and a PTH response gene.^(19)^ Deletion of *Tgif-1* leads to reduced osteoblast differentiation *in vitro* and bone remodeling *in vivo*. Calvarial osteoblasts treated with PTH have increased expression of *Tgif-1*. Bone turnover in iPTH treated *Tgif-1*^-/-^ mice is reduced relative to wildtype controls as well as a decreased response in the trabecular bone. The authors identified a binding site for activator protein 1 (AP1) family members in the promoter region of *Tgif-1*. Following PKA activation and CREB phosphorylation, AP1 is activated. When the *Tgif-1* gene was truncated to lose the AP1 binding site, reporter gene activation in a luciferase assay was abolished. This paper indicates that PTH activates *Tgif-1* on the PKA, cAMP, and AP1 axis.

ATF4, a transcription factor critical for proliferation, differentiation, and survival in osteoblasts, is a downstream and critical mediator of PTH signaling. In the absence of *Atf4*, anabolic effects of iPTH were attenuated^(20)^ with *Atf4*^-/-^ mice having a lower bone volume, trabecular number, trabecular thickness and mineral apposition rate than wildtype mice. Additionally, there was an increase in osteoblast apoptosis, while osteoblast maturation and differentiation were inhibited (as measured by *Osterix* expression). These data suggest that ATF4 is an important factor for the full anabolic effect of iPTH.

The protooncogene c*-fos* is an early response gene that increases rapidly after PTH administration *in vitro* and *in vivo.*^(21)^ Levels of c-Fos affect both osteoclasts and osteoblasts. As a member of the AP1 transcription factor family, c-Fos is integral to a variety of functions and knockout mice have an osteopetrotic-like phenotype, characterized by lack of osteoclasts ^(22)^. Not only was there a lack of anabolic actions of iPTH in growing *c-fos^-/-^* mice but there was actually an anti-anabolic effect with decreased bone mineral density, femur calcium content, and vertebral bone volume. Transplantation studies further implicated hematopoietic cells in the bone microenvironment as critical for anabolic actions of iPTH.^(23)^

Investigation into actions of iPTH focus on the osteogenic genes that favor osteoblast differentiation. However, there are also players involved in negative feedback. One of these downstream targets of iPTH is nuclear matrix protein 4/cas interacting zinc finger protein (Nmp4/CIZ) that acts as a transcriptional attenuator in osteoblasts. Several studies have shown that with overexpression of Nmp4/CIZ, MC3T3-E1 cells suppress expression of osteogenic genes that can be induced with PTH or BMP2.^(24,25)^ Using a loss of function mutation in *Nmp4/Ciz*, Robling et al. found that iPTH anabolic actions were enhanced in trabecular bone seen in the femur increasing 2.906-fold in *Nmp4/Ciz*-null mice relative to the wildtype.^(26)^ Cortical bone was not impacted in terms of response to iPTH, with bone mineral density and bone mineral content responding similarly in control and deficient mice. The response of osteoblasts to iPTH is dependent on their bone microenvironment location in femurs. Follow-up studies in the same model confirmed the trabecular-specific enhanced response in vertebrae and tibiae with seven weeks of iPTH treatment. More specifically, the number of osteoclasts was increased in the *Nmp4/Ciz*-null mice without treatment, but relatively suppressed with PTH treatment. Childress et al. hypothesized that Nmp4/CIZ impacts both osteoblast and osteoclast responses to PTH.^(27)^ In order to better understand the anabolic window, the same group treated the *Nmp4/Ciz-*null mice for three weeks.^(28)^ Again, the anabolic response was increased, with femoral trabecular bone volume increasing 2.523-fold compared to the response in wildtype mice. Additionally, bone marrow cells had increased osteoprogenitor potential, suggesting that Nmp4/CIZ limits the anabolic response by restricting progenitor cell response to iPTH.

β-arrestins are a family of cystosolic proteins that are triggered by activation of seven transmembrane receptors, including PTH1R, where they play a central role in desensitization.^(7)^ In response to PTH(1-34), β-arrestin1 and β-arrestin2 are translocated to the plasma membrane, where there is an association of the receptor with β-arrestins, and internalization of the receptor-β-arrestin complexes. Skeletal PTH effects are modulated by β-arrestin2, as first reported in 2005.^(29)^ β-arrestin2 mediates rapid uncoupling of PTH1R from the G protein.^(30)^ In the vertebrae of *β-arrestin2*^-/-^ mice, the anabolic effect of iPTH was lower than wildtype.^(29)^ Getsy-Palmer et al. also found a diminished iPTH effect in the lumbar and tibia of *β-arrestin2*^-/-^ mice.^(31)^ It is unknown if these results can be attributed to exaggerated G protein signaling stimulating osteoclastic bone resorption or the loss of β-arrestin2-mediated signals from PTH, or both.

PTH activates p38α mitogen activated protein kinase (MAPK) in osteoblasts, which can be modulated by β-arrestins.^(32)^ The impact of losing p38α under the control of the osteocalcin promoter on anabolic iPTH was studied by Thouverey and Caverzasio.^(33)^ In these mice, the femoral trabecular bone volume was increased with iPTH, but to a lesser extent than the response in control mice. Trabecular mineral apposition rate, number of osteoblasts, mineralized surface, and apoptotic osteoblast response to iPTH were all attenuated in mice lacking p38α in cells expressing osteocalcin. This demonstrates that the p38α MAPK activity is essential in mature osteoblasts and osteoclasts for iPTH anabolic actions.

AMP-activated protein kinase (AMPK) is another kinase involved in bone homeostasis. AMPK has three subunits: the catalytic α and regulatory β and γ subunits. Once it is activated, AMPK generates ATP through a catabolic pathway and inhibits cellular pathways that consume ATP.^(34)^ The AMPKα1 subunit is the dominant form in bone, and AMPK activators stimulate osteogenesis *in vitro.*^(35)^ Therefore, the potential role of AMPKα1 in anabolic actions of PTH is of interest. In ovariectomized *Ampkα1^-/-^* mice, there is a dampened anabolic response to iPTH in tibial trabecular bone volume.^(36)^ However, when mice were not ovariectomized, the *Ampkα1*^-/-^ mice had an increased anabolic response to iPTH (4.250-fold change in trabecular bone volume). This could be due to a lesser bone loss response to ovariectomy in the knockout mice than the wildtype mice, resulting in the bone responding differently. This data suggests that AMPKα1 mediates the effects of iPTH on bone turnover, but is not necessary for an anabolic response.

Mitogen-activated protein kinases (MAPKs) are downstream of PTH1R activation, and can regulate multiple osteoblast activities.^(37-39)^ They also play an important role for Runx2 phosphorylation and activity.^(40)^ MAPKs are regulated by (de)phosphorylation to be (in)activated by several enzymes, including MAPK phosphatases (MKPs), a family of 11 members. MKP1 is activated by PTH or PTHrP in osteoblasts and plays a role in osteoclasts and anti-inflammatory bone loss. *Mkp* knockout mice were treated with iPTH to understand how Mkp1 regulates the anabolic response.^(41)^ In the femur of female mice, iPTH was attenuated in *Mkp^-/-^* compared to wildtype as indicated by trabecular number, trabecular spacing, and trabecular thickness; however, the bone volume was modestly increased with a fold change of 1.250. Together, these data suggest that *Mkp* may be required for the full anabolic response to PTH.

Neurofibromin is a GTPase-activating protein (GAP) that affects generation of MAPK, as well as Ras, resulting in increased ERK activity. Up to half of neurofibromatosis type 1 (Nf1) patients exhibit skeletal pathology. In an *Nf1* haploinsufficient mouse, osteoprogenitor proliferation is increased, differentiation is decreased, and apoptosis is premature.^(42)^ Therefore, it was hypothesized that iPTH in *Nfp^+/-^* mice would result in an impaired skeletal anabolic response.^(43)^ *In vivo* results with iPTH showed that heterozygous mice responded similarly as wildtype mice in gaining bone volume. In *Nf1^+/+^* wildtype mice, iPTH increased trabecular number whereas in *Nf1^+/-^* mice iPTH increased trabecular thickness. Authors postulated that this was due to activation of different pathways since the heterozygous mice have an activated Ras pathway and a reduced PKA pathway or different osteoclastic and osteoblastic function in the wildtype versus haploinsufficient mice.

The transcription factor Runx2 is a well-accepted regulator of osteoblastogenesis, referred to as the master gene for osteoblasts.^(44)^ Mice with homozygous mutations in *Runx2* are lethal at birth, with skeletons that do not ossify.^(45)^ In transgenic mice overexpressing *Runx2* in osteoblasts (using the type I collagen promoter), mice were viable, but maturation of osteoblasts was inhibited and bones were osteopenic.^(46)^ After six weeks of iPTH treatment, *Runx2* transgenic mice had a decreased anabolic response compared to wildtype mice as measured by bone volume, trabecular thickness, osteoclast number, cortical thickness, mineral apposition rate, mineralizing surface, and bone formation rate.^(47)^ High levels of Runx2 abolished the anabolic response to PTH, and validates the critical role that osteoblast differentiation stage has on PTH actions since PTH1R expression varies with osteoblast differentiation stage.^(48)^

*Summary:* Anabolic PTH requires PTH1R receptor availability, and specifically the Gα_s_ subunit. Several models that affect receptor availability and function through different mechanisms, including phosphorylation and recycling support this. Furthermore, models have been used to analyze signaling downstream of PTH1R and report that there are several important proteins at various levels of signaling cascades, including well known osteogenic players.

***Downstream Mediators***

Fibroblast Growth Factor (FGF)

Fibroblast growth factor-2 (FGF2) is a key molecule for bone formation and maintenance of bone mass under homeostatic conditions.^(49)^ FGF2 stimulates proliferation and impairs apoptosis of osteoblast precursors. PTH treatment of MC3T3-E1 cells increases *Fgf2* mRNA expression^(50)^, and FGF2 treatment of bone marrow stromal cells increases *Pth1r* mRNA expression.^(51)^ Hence, it is not surprising that the anabolic response of iPTH was attenuated in *Fgf2^-/-^* mice.^(52)^ Femoral μCT analysis of iPTH treatment in mice for four weeks showed an increased bone volume, increased trabecular number, and decreased trabecular spacing in *Fgf2*^+/+^ but not *Fgf2*^-/-^ mice. In concert with the animal studies, osteoporotic patients treated with iPTH had increased serum FGF2.^(53)^ In an attempt to elucidate the mechanism behind the murine phenotype, Sabbieti et al. treated calvarial osteoblasts from *Fgf2^+/+^* and *Fgf2^-/-^* mice with PTH.^(54)^ Results support involvement of FGF2 in the anabolic iPTH response via RUNX2 activation of CREB-phosphorylation. Later studies found that iPTH treatment mediated FGF2 at least in part via the transcription factor ATF4.^(55)^ Other mediators of this response have also been explored. Xiao et al. found that Wnt signaling is involved in iPTH treatment of *Fgf2*^-/-^ mice.^(56)^ More specifically, Wnt10b was not increased and Wnt antagonist was increased in mutant mice in response to iPTH; both results were unlike the wildtype mice, and implicate Wnt signaling in anabolic actions of PTH in bone.

FGF receptor 3 (FGFR3) is one of the major receptors for FGF2 on osteoblasts, and

*Fgfr3^-/^*^-^ mice are osteopenic associated with reduced cortical bone thickness and defective trabecular bone mineralization.^(57)^ In these mice, iPTH was anabolic and comparable to wildtype mouse phenotypes, indicating that *Fgfr3* is dispensable for this response.^(58)^ Gain-of-function murine mutations in *Fgfr3* mimic clinical presentations of achondroplasia, including increased expression of osteocalcin and osteopontin.^(59)^ When these mice were administered iPTH, their anabolic response was enhanced relative to wildtype control mice.^(60)^ This was indicated by a 2.814-fold increase in the trabecular bone volume of the femur following four weeks of treatment. Although a reduction of *Fgfr3* was not necessary for anabolic iPTH, an abundance of *Fgfr3* activation bolstered the iPTH anabolic response.

FGF23, another member of the fibroblast growth factor family, regulates phosphate homeostasis on an axis with PTH and 1,25(OH)_2_ vitamin D. FGF23 is secreted by osteoblasts and osteocytes into circulation and binds to a complex in the kidney that includes Klotho (*KI*). This results in increased phosphate excretion when levels are too high. FGF23 can also target the parathyroid gland as a negative feedback regulator of PTH. PTH has also been shown to stimulate FGF23.^(61)^ Young *Fgf23*^-/-^ or *Kl*^-/-^ mice were administered iPTH and their bone phenotypes were analyzed.^(62)^ Anabolic actions of iPTH were comparable in both mutant and wildtype mice, including trabecular bone volume, suggesting *Fgf23* is also dispensable for anabolic actions of iPTH.

Wingless-related Integration Site (Wnt)

Wingless-related integration site (Wnt) signal transduction pathways involve a large family of Wnt proteins, regulators, and downstream effectors. Wnt proteins bind to a Frizzled family receptor, leading to changes in gene expression and impact processes from development and patterning to cell fate, proliferation, and migration. Canonical Wnt signaling is important for regulation of osteoblasts and bone formation.^(63)^

Patients with a heterozygous *Wnt1* mutation (p.R235W) present clinically with early onset osteoporosis.^(64)^ A mouse model was developed with the same mutation in the Wnt1 ligand to analyze the bone.^(65)^ In 12 week old *Wnt1^R235W/R235W^* mice, there was a lower bone formation rate, resulting in reduced trabecular and cortical bone mass. Female, 52 week old *Wnt^+/+^* and *Wnt1^+/R235W^* were treated for four weeks with daily injections with 80 μg/kg/day hPTH(1-34). Both genotypes responded anabolically to PTH as reported in the vertebral, trabecular, and periosteal bone formation rates, serum procollagen I N-terminal peptide (PINP) and C-terminal telopeptide (CTx), and cortical thickness.

Low-density lipoprotein receptor-related protein 5 (LRP5) is a co-receptor for Wnt signaling. Loss of function mutations are associated with the human disease osteoporosis-pseudoglioma syndrome (OPPG), an autosomal recessive disease with reduced bone mass and strength. iPTH treatment in *Lrp5^-/-^* mice was fully anabolic, with femoral bone mineral density increasing similarly in *Lrp5^+/+^* and *Lrp5^-/-^* mice.^(66)^ Using a different strain of *Lrp5^-/-^*, Iwaniec et al. studied PTH with 80 μg/kg/day hPTH(1-34) on alternate days for 6 weeks.^(67)^ Knockout mice had a similar anabolic response to treatment, with the number of osteoblasts per surface, bone formation rate, bone mineral content, and bone mineral density all increased with PTH. Both of these studies agree that *Lrp5* is not essential for the anabolic response to PTH.

Low-density lipoprotein receptor-related protein 6 (LRP6) has overlapping functions with LRP5. However, LRP6 in osteoblasts is required for the anabolic actions of iPTH. Using a conditional mutant model driven by the human osteocalcin promoter, the anabolic response in femoral bone volume was a -1.255-fold change compared to the wildtype response.^(68)^ Of note, loss of *Lrp6* from mature osteoblasts did not alter bone development, but did block remodeling with iPTH treatment. In a follow up study, the same group showed that iPTH did not suppress *Sost* mRNA expression in bone and the number of SOST positive osteocytes were similar in wildtype littermate controls.^(69)^ Therefore, they concluded that LRP6 is required for iPTH suppression of *Sost*, a mediator to inhibit osteoblastogenesis and bone formation.

Downstream of the receptor-ligand interaction in canonical Wnt signaling is β-catenin (βcat), which acts as an intracellular signal transducer. In the *Lrp6* null mice treated with iPTH, induction of *βcat* failed.^(70)^ Using the 10 kb-DMP1 promoter, *βcat* was conditionally knocked out in osteoblast and osteocyte cells in mice.^(71)^ They were treated with tamoxifen to induce recombination of the floxed *βcat* gene in cells expressing DMP1 starting at 12 weeks old and three days later, iPTH treatment was administered for five weeks. In these studies, iPTH treatment increased the bone volume of the femur (2.115-fold) and lumbar vertebrae (2.517-fold) more than non-transgenic, tamoxifen treated mice. However, DEXA measurements were inconsistent and show that BMD did not increase with PTH treatment when *βcat* was deleted from *Dmp1*-expressing cells. The representative data for resorption (number of osteoclasts/bone surface, serum CTX, cortical porosity) all suggested that it was further increased with iPTH and tamoxifen treated transgenic mice. The authors concluded that iPTH increased bone formation whether or not *βcat* is present in DMP1 expressing cells, but loss of *βcat* in those cells significantly increased resorption. In order to further elucidate canonical Wnt signaling and PTH mechanisms, floxed *βcat* mice were crossed with osterix-cre transgenic mice.^(72)^ With this model, *βcat* is ablated from cells expressing osterix (osteoblasts and a few mesenchymal stromal cells) when exposure to doxycycline is terminated. iPTH had blunted anabolic actions when *βcat* was ablated from osterix-expressing cells in the femur. The serum bone formation marker P1NP was not increased in response to iPTH in *βcat* mutant mice, although it was in control mice. In both groups of mice, the resorption marker CTX increased. These findings suggest that *βcat* in osterix-expressing cells is necessary for the anabolic response to iPTH.

Secreted frizzled-related protein 1 (sFRP-1) is a Wnt antagonist, binding to the Wnt ligand. Ablation of *sFRP-1* in adult mice leads to increased trabecular bone that is attributed to increased osteoblast proliferation, differentiation, and function and decreased apoptosis.^(73)^ Using female *sFRP-1^+/+^* and *sFRP-1^-/-^* mice, Bodine et al. studied the effect of iPTH over 30 days in three age groups: starting at 8, 24, or 36 weeks of age.^(74)^ At all the timepoints, there was an anabolic response to PTH albeit less in the knockout than that of the wildtype. Comparing the femoral bone volume response to iPTH, *sFRP-1^-/-^* mice had a 0.711, 0.627, and 0.332-fold change compared to wildtype in anabolic response after treatment of 8, 24, and 36 week old mice respectively. Using a transgene model to over-express *sFRP-1*, Yao et al. found mutant mice had attenuated PTH responses.^(75)^ However, this was only after two weeks of treatment in 12-14 week old mice (40 μg/kg/day hPTH(1-34) for 5 days/week) and confined results to the rapid effects of iPTH. Taken together, sFRP-1 is necessary for the full anabolic actions of iPTH with longer treatment in aged mice.

Dickkopf 1 (DKK1) is an extracellular inhibitor of Wnt signaling that interacts with LRP5/6 to block the formation of a receptor complex with Frizzled and Wnt ligand. *In vivo* models suggest that *Dkk1* suppresses bone formation. In osteoblast cultures treated with PTH, *Dkk1* expression is inhibited. Yao et al. used transgenic mice overexpressing murine *Dkk1* under the 2.3-kb rat collagen α-1 promoter, expecting that iPTH would not produce an anabolic response without the suppression of *Dkk1*.^(76)^ Transgenic bone mass and histomorphometric parameters of bone formation were reduced compared to wildtype. When treated with iPTH, bone mineral density of the spine, femur, and total body showed similar levels between wildtype and transgenic mice. The number of osteoblasts was significantly increased in both genotypes, but with a more modest increase in transgenic mice, while the number of osteoclasts were increased in response to iPTH in the wildtype mice, but not in the transgenic mice, indicating decreased bone turnover. However, overexpression of *Dkk1* does not attenuate the anabolic response in bone to iPTH.

Sclerostin (SOST) is a potent bone formation inhibitor, secreted by osteocytes to block Wnt signaling by binding directly to LRP5 and LRP6. Accordingly, *Sost* knockout mice have a high bone mass phenotype while *Sost* transgenic mice have a low bone mass phenotype. Additionally, iPTH directly suppresses *Sost* expression in osteocytes.^(77)^ iPTH-induced bone anabolism was attenuated (0.391-fold difference comparing wildtype femoral bone volume) in mice with a *Sost* transgene.^(78)^ iPTH has also been introduced to *Sost^-/-^* mice, in which treatment did not result in as great an anabolic effect in bone as found in wildtype control mice. Robling et al. presented data from male *Sost^-/-^* or *Sost^+/+^* mice treated with 0, 30, or 90 μg/kg/day of iPTH for six weeks beginning at ten weeks of age.^(79)^ In these experiments, iPTH had reduced capacity to increase bone mineral density and bone mineral content. Additionally, bone volume for the femur was changed by 0.779- and 0.877-fold using 30 and 90 μg/kg/day of iPTH, respectively. Further histomorphometric analysis of the femur showed that the bone formation rate on the periosteal surface and endocortical surface of bone responded in an anabolic manner to the iPTH. However, cortical porosity increased, which affects the bone mineral density and bone mineral content values. This suggests that the response of iPTH requires *Sost*, but in a bone location-dependent manor.

*Sost* expression can also be modulated by HDAC4 and HDAC5, two class IIa histone deacetylase enzymes. When dephosphorylated, HDAC4 and HDAC5 translocate to the nucleus and block *Sost* expression through the transcription factor MEF2.^(80)^ These genes are required for iPTH-induced *Sost* expression in osteocytes, but not for anabolic actions of PTH.^(81)^ Wein et al. treated *HDAC4^fl/fl^*;DMP1-cre, *HDAC5^-/-^*, and *HDAC5^-/-^*;*HDAC4^fl/fl^*;DMP1-cre mice with iPTH. In respect to bone volume, the response in *HDAC4^fl/fl^*;DMP1-cre was comparable to wildtype controls. *HDAC5^-/-^* and *HDAC5^-/-^*;*HDAC4^fl/fl^*;DMP1-cre mice had a slight increased anabolic response to iPTH. Data indicates that while these genes are impacting the bone response to iPTH, there are also other regulators at play.

N-cadherin (*Cdh2*) is a negative regulator of canonical Wnt signaling, and abundant in osteogenic cells. βcat links cadherins to the actin cytoskeleton adherens junctions, regulating intracellular signaling networks.^(82)^ By studying *Cdh2* knockout mice with ablation specific to osterix positive cells, Revollo et al. evaluated their response to iPTH.^(70)^ The *Cdh2* conditional knockout mice had an accentuated trabecular bone response, with bone volume increased 3.815-fold compared to anabolic controls. In addition, the bone mineral density and trabecular thickness increased more in the conditional knockout, and the trabecular number decreased less. These results indicate that *Cdh2* regulates iPTH responsiveness in bone. *Cdh2* was also conditionally knocked out of DMP1 expressing cells, which is specific to osteocytes.^(83)^ These mice had a greater anabolic response to iPTH, with a bigger increase in bone volume than control mice. This study also focused on the regulating effects of PTH on SOST, which had enhanced suppressive effects in conditional *Cdh2* knockout mice. They hypothesized that this occurs via LRP6, with increased availability to respond to PTH.

Bone Morphogenetic Proteins (BMP)

Bone morphogenetic proteins (BMPs) are a family of growth factors that contribute to orchestration of bone and cartilage formation during development and healing. BMP2 and BMP7 are used clinically to enhance bone healing, and the effect of these proteins in bone remodeling was more clearly defined using iPTH.^(84)^ Using a tamoxifen induced system (R26CreER), BMP2 and BMP4 were bred as double-conditional knockouts, and treated with tamoxifen for five days. Mice were also ovariectomized or sham operated before iPTH treatment. BMP2 and BMP4 double knockout mice had reduced bone compared to controls and when ovariectomy was performed, the control and double knockout mice lost bone similarly. However, only the control mice demonstrated an anabolic response to iPTH. Hence, BMP2 and BMP4 are required for the trabecular anabolic effects of iPTH.

Insulin-like Growth Factor (IGF) and Growth Hormone (GH)

PTH treatment increases osteoblast production of insulin-like growth factor I (IGF-I), which has long been implicated in the anabolic actions of iPTH. Of note, growth hormone (GH) also stimulates IGF-I production from osteoblasts, therefore the age of the mice when evaluating this model is an important consideration. When IGF-I was globally deleted, the anabolic actions of iPTH were lost in growing mice (five weeks old, 160 μg/kg/day iPTH) ^(85)^ and adult mice (15 weeks old, 80 μg/kg/day iPTH).^(86)^ This supports the dependence on IGF-I for the anabolic response of bone to iPTH.

Adult female mice were used to assess iPTH anabolic actions in a congenic 6T mouse that has lower circulating IGF-I (about 30-50% reduction) compared to wildtype controls. ^(87)^ These mice had an impaired anabolic bone response to iPTH, further validating the necessity of IGF-I for iPTH effects. In a complimentary study, mice carrying a hepatic IGF-I transgene (HIT) were analyzed for their response to PTH.^(88)^ This model has a three-fold increase in serum IGF-I levels, albeit normal tissue expression. Knockout-HIT mice maintain their increase in serum IGF-I, but have blunted expression in tissue. HIT mice had an augmented response to iPTH not seen in the knockout-HIT mice, supporting their synergistic relationship and that PTH is dependent on tissue IGF-I.

Yakar et al. used three different genetic manipulations to study the mechanism of IGF-I during iPTH treatment.^(89)^ These included: a liver specific deletion of IGF-I, a global deletion of the acid-labile gene (ALS, part of the tertiary complex of circulating IGF-I), and both liver knockout of IGF-I with ALS inactivation. Endocrine-acting IGF-I originates in the liver, and circulates in a complex that includes ALS. The combination of these three models evaluates the effect of circulating IGF-I as opposed to that acting via an autocrine/paracrine route. This was done because serum IGF-I positively correlates with bone mineral density and inversely correlates with fracture risk. With the outcome variable of vertebral bone volume, the liver specific deletion had a 2.150-fold increase, the ALS knockout had a -0.300-fold decrease, and the double knockout mice had a -0.350-fold decrease compared to the anabolic response to iPTH in wildtype mice. The strains with deficient ALS, which have 65% decreased circulating IGF-I, either alone or in combination with deficiency of liver-derived IGF-I, had lesser anabolic response despite similar numbers of osteoblasts and osteoclasts. This suggests that the liver-derived IGF is an important mediator of iPTH in bone.

Pregnancy-associated plasma protein-A (PAPP-A) cleaves an inhibitor of IGF action, enhancing local IGF effects, but not circulating IGF-I. IGF expression is increased in response to iPTH, but not in PAPP-A knockout mice.^(90)^ Accordingly, the anabolic effect of iPTH was also attenuated in PAPP-A deficient mice. Interestingly, this effect was in cortical bone, not trabecular, suggesting IGF signaling is more pronounced in cortical bone.

IGF-I binds to its receptor (IGF-IR) on osteoblasts, promoting proliferation, differentiation, and survival. Global IGF-IR knockout mice have low survival, and hence the receptor has been deleted specifically in mature osteoblasts using cre recombinase under an osteocalcin promoter.^(91)^ The conditional IGF-IR knockout mice have less trabecular and cortical bone at baseline, and a dampened effect of anabolic PTH treatment which was more apparent in the primary spongiosa compared to the secondary spongiosa. Wang et al. suggest that this is due to higher expression of IGF-I in those osteoblasts.^(92)^ IGF-I regulates expression of ephrin B2 (EphB2) in osteoblasts and osteoclasts and ephrin B4 (EphB4) in osteoblasts. These genes are involved in osteoblast differentiation, and deletion of IGF-IR in osteoblasts decreases expression of EphB2 and EphB4, and blunts osteoblast differentiation with anabolic PTH.^(91)^ Treatment of wildtype mice with soluble EphB4, which is an inhibitor of EphB2 and EphB4, resulted in a blunted anabolic response to iPTH.^(93)^ μCT and histomorphometric analysis of male mice show decreased bone volume, decreased trabecular number, and increased trabecular spacing despite similar increases in mineral apposition rate and bone formation rate. There was an increase in osteoclast number and bone surface coverage in mice treated with iPTH in combination with EphB4 versus iPTH alone. These data support that ephrin B2 and B4 are critical for the ability of osteoblasts to support osteoclast formation.

IGF-I binds to tyrosine kinase receptors, which then activate adapter molecules insulin receptor substrate (IRS) 1 and 2. IRS-1 and IRS-2 are expressed in bone, and deleting these genes results in osteopenic bone phenotypes. However, *Irs-1^-/-^* mice exhibit decreased bone formation and resorption (i.e., low bone turnover) ^(94)^ and *Irs-2^-/-^* mice have decreased bone formation and increased resorption.^(95)^ iPTH actions on bone are conserved in *Irs-2^-/-^*, but suppressed in *Irs-1^-/-^* mice.^(96)^ *Irs-1^-/-^* versus wildtype mice have a decreased anabolic response as measured by bone density of the femur, tibia and vertebrae. *Irs-1* is only expressed in osteoblasts, whereas *Irs-2* is expressed in osteoblasts and osteoclasts. Comparing these models in the same study revealed that *Irs-1*, not *Irs-2* mediates anabolic actions of PTH.

IGF-I and GH participate in the same axis, with IGF-I production stimulated in the liver by GH. It is well-accepted that GH affects the development of the long-bone diaphysis and the trabecular bone compartment.^(97)^ GH receptor (GHR) was studied to understand how osteocytes integrate the GH/IGF axis to enhance skeletal mineralization.^(98)^ After puberty, control mice increased PTH levels as expected. However, *DMP1-cre;GHR^f/f^* mutant mice did not experience this increase, pointing to a relationship between GHR and iPTH. Liu et al. went on to evaluate the effect of iPTH in these mice during puberty (four to eight weeks old). The mutant mice had a blunted anabolic iPTH effect, with a 0.234-fold change in the response as measured by trabecular bone volume of the femur. In addition, trabecular number and thickness and mineral apposition rate were not increased in the *DMP1-cre;GHR^f/f^* mice as they were in controls. Taken together, the *DMP1-cre;GHR^f/f^* mice responded poorly to iPTH, and suggest that GHR is an important factor in anabolic PTH actions.

Epidermal Growth Factor (EGF)

In osteoblasts, epidermal growth factor receptor (EGFR) stimulated proliferation and inhibited differentiation.^(99)^ PTH increased the expression and activity of EGFR in osteoblasts but the role of EGFR in anabolic PTH mechanisms was unclear. Schneider et al. used an *in vivo* model to address this in Waved-5 (Wa5) mice that have an antimorphic *Egfr* allele that acts as a dominant negative receptor.^(100)^ *Egfr* knockout mice could not be used because they are not viable. After four weeks of iPTH, Wa5 mice had an insignificant decrease in vertebral response compared to littermate controls, as measured by cortical, trabecular, and total bone mineral density. Femur analysis also revealed a slightly dampened anabolic response with respect to bone area, bone formation rate, bone volume, and cortical area. Reduced EGFR signaling did not blunt the anabolic actions of iPTH.

Amphiregulin (AREG) is a ligand of epidermal growth factor receptor, and a downstream target of PTH.^(101,102)^ AREG stimulates proliferation and prevents differentiation and mineralization of osteoblasts. Upon stimulation with PTH *in vitro*, osteoblasts release AREG that acts on epidermal growth factor receptors in mesenchymal progenitors.^(103)^ Global *Areg* knockout mice were administered iPTH to have a full understanding about how it is involved in bone formation and turnover.^(104)^ Markers of anabolic iPTH were similar in wildtype and *Areg* knockout mice, including bone mineral density, cortical thickness, bone formation, and bone volume. There was a reduction in osteoclast numbers in the *Areg^-/-^* mice suggesting that it may play a role in osteoclast recruitment or maturation in response to iPTH. Overall, these findings suggest that AREG is not necessary for anabolic iPTH actions.

*Summary:* Within the FGF family, FGF2 is indispensable for the anabolic response of iPTH, while others, such as FGFR3 and FGF23, are not required, indicating potential for compensation. In regard to Wnt signaling, receptor LRP6, but not LRP5, is required for the anabolic response. Wnt antagonists are necessary for the anabolic response, however more studies to elucidate the pathways are necessary. There are likely repetition and compensation mechanisms, leading to complex relationships in signaling activation. Multiple studies implicate the importance of IGF-1 in anabolic iPTH, and specifically liver-derived IGF-1, via downstream EphB2, EphB4, and Irs-1, and upstream GHR.

***Cell Regulatory Factors***

Apoptosis

Understanding apoptosis in bone turnover is a strategic approach aimed at understanding how to prolong the life of bone forming osteoblasts and reduce bone resorbing osteoclasts for osteoporotic therapies.^(105)^ Apoptosis of myeloid, lymphoid, and neuronal lineages is suppressed by B-cell leukemia/lymphoma 2 (*Bcl2*). *Bcl2^-/-^* mice are smaller than littermates, although skeletal proportions are intact.^(106)^ Yamashita et al. reported that osteoblast function is not affected by ablation of *Bcl2*, but osteoclast number and serum TRACP 5b levels were lower in knockout mice.^(107)^ Growing *Bcl2^+/+^* or *Bcl2^-/-^* mice were administered iPTH for 9 days (the time frame was limited by the viability of *Bcl2^-/-^* mice, which have compromised kidney function). Tibial μCT analysis with iPTH treatment showed increased bone volume in both genotypes. The osteoblast number was also increased with iPTH, regardless of genotype, consistent with a normal anabolic response to iPTH. Results indicated the *Bcl2* is dispensable for the skeletal actions of iPTH. BCL2 interacting mediator of cell death (BIM, aka BCL2L11) interacts with Bcl2 as an apoptotic activator. In order to study *Bcl2^-/-^* skeletal biology in adult mice, they can be crossed with *Bim^+/-^* to survive beyond 6 weeks, resulting in *Bcl2^-/+^Bim^+/-^* and *Bcl2^-/-^Bim^+/-^* mice.^(108)^ Contrary to the model described above in *Bcl2^-/-^* mice, osteoclasts from *Bcl2^-/-^Bim^+/-^* exhibited normal resorption but osteoblasts had impaired mineralization capacity. In the double-heterozygous, control mice, femoral bone mineral density increased with four weeks of iPTH treatment. However, the *Bcl2^-/-^Bim^+/-^* mice did not have an anabolic response, with a reduction in the iPTH-induced bone mineral density. These two studies analyzing iPTH in *Bcl2* knockout mice utilized different ages – a few days old vs. weeks old – which could account for the differential results. Contributions from the different genotypes should also not be dismissed.

Immunity

The receptor for advanced glycation end products (RAGE) is a transmembrane receptor part of the immunoglobulin family, with multiple ligands. It is implicated in osteoclast activation and bone remodeling. *RAGE*^-/-^ mice have increased bone mineral density and bone mass, as well as reduced osteoclast activity.^(109)^ When treated with iPTH, cortical bone responds in a similar anabolic fashion in both wildtype and *RAGE* knockout mice. However, trabecular bone volume was decreased with seven weeks of treatment in *RAGE*^-/-^ mice compared to the expected anabolic actions found in wildtype mice.^(110)^ Histomorphometric analysis was not performed in this study, however the femurs were analyzed for gene expression. Many osteoblast-mediating genes were decreased in the *RAGE* null mice, but stimulated in similar proportions as wildtype in the iPTH treated groups. RAGE is necessary for full anabolic iPTH responses, but further study is necessary to elucidate how deletion of RAGE exerts its inhibitory action of anabolic responses to iPTH.

*Pth1r* is expressed not only on osteoblasts but also on T cells, and these cells are responsive to iPTH and stimulate osteoblast differentiation.^(111)^ *Pth1r* deletion in T cells, using the *Lck* reporter, resulted in a blunted anabolic response to iPTH by blocking iPTH-induced Wnt10b production by T cells.^(112)^ Trabecular bone volume showed a reduced anabolic response to iPTH in this PTH1R T cell deficiency model in both young and mature mice. Cortical bone in wildtype and deficient mice responded similarly to iPTH treatment. This work highlights an interaction between iPTH signaling in bone and immune cells.

Wnt10b production by T lymphocytes has also been linked to anabolic actions of iPTH by Terauchi et al.^(113)^ First, mice lacking αβ T cells (*TCRβ*^-/-^) were treated with iPTH and experienced a blunted anabolic response (0.503-fold change in trabecular bone volume). Next, to characterize the subset of T cells required for iPTH actions, mice that were null for *MHC I*, *MHC II*, or both *MHC I* and *MHC II* were used. The reduction in trabecular bone response to iPTH in *MHC I*^-/-^ mice was about 0.173-fold, whereas *MHC II*^-/-^ had a minimal effect (1.038-fold change), indicating that CD8 T cells are involved with anabolic actions with PTH treatment. Data also support Wnt10b as a key signaling ligand in this cascade. *Wnt10b* expression was induced in bone marrow of wildtype iPTH treated mice, but not in *TCRβ*^-/-^ iPTH treated mice. Additionally, wildtype and *TCRβ*^-/-^ mice were subjected to adoptive transfer of T cells that either express or do not express *Wnt10b*. Following iPTH treatment in these mice, the anabolic response was only recovered in the *TCRβ*^-/-^ mice that had T cells expressing *Wnt10b*. Therefore, activation of Wnt by T cells is a crucial pathway for the anabolic actions of iPTH treatment.

To understand if anabolic actions of iPTH are mediated by interactions of T cells and stromal cells, Robinson et al. studied CD40L (also known as CD154).^(114)^ CD40L mediates T cell interactions with stromal cells by binding to CD40 and several integrins. CD40L has been studied in skeletal maturation, due to the low bone density found in humans with a mutation affecting CD40L production.^(115)^ In *CD40L*^-/-^ mice treated with iPTH, the anabolic response according to femoral bone volume was decreased by 0.135-fold. The mineral apposition rate and bone formation rate were also decreased. *Wnt10b* activation in response to iPTH was also decreased when CD40L was not present. To understand if the blunted anabolic response to iPTH was due to CD40L from T cells, mice lacking *TCRβ* were engrafted with T cells from *CD40L*^+/+^ and *CD40L*^-/-^ mice. Mice given the *CD40L*^+/+^ T cells had a normal response to iPTH, while those given *CD40L*^-/-^ T cells still had reduced bone volume, confirming that CD40L specifically from T cells is necessary for a full anabolic response, highlighting the importance for T cell stimulation of osteoblasts.

The T regulatory (Treg) cell compartment is a suppressive immune cell population defined by their expression of transcription factor *Foxp3*. Treg cells are involved in bone activity, including blunted bone resorption, preventing ovariectomy-associated bone loss, and regulating osteoclast formation.^(116)^ Teriparatide treatment in humans increased Treg cells in peripheral blood, and iPTH in wildtype mice increased the number of Treg cells in the bone marrow.^(117)^ In order to understand if increasing Treg is required for the anabolic response of iPTH, expansion of this population was partially prevented by treating mice with a CD25 antibody. In this model, the frequency of bone marrow Tregs was depleted. The anabolic response to iPTH was attenuated as seen in trabecular and cortical bone, mineral apposition rate, bone formation rate, and the number of osteoblasts in CD25 antibody treated femurs. A second model to deplete Treg cells, DEREG mice, was used in which the diptheria toxin receptor is expressed under the control of *Fox3p*. Upon delivery of diptheria toxin, *Fox3p* expressing Treg cells, are depleted. This model confirmed that Treg cells are required for the anabolic response of iPTH. DEREG mice treated with diptheria toxin did not have an anabolic response, with lower changes in trabecular bone volume, mineral apposition rate, bone formation rate, and number of osteoblasts. Based on these two models, Yu et al. concluded that Treg cells are required for anabolic iPTH effects since blocking the expansion of this population also attenuated anabolic bone actions via iPTH.

T cell function with iPTH has also been linked to the microbiota.^(118)^ Li et al. identified that germ-free mice had a decreased response to anabolic iPTH as measured by trabecular bone volume, mineral apposition rate, bone formation rate, and osteoblast and osteoclasts on the bone surface. The microbiota depletion was also associated with lowered butyrate levels, and when those levels were brought back to physiological ranges, the mice were able to respond normally to iPTH. Butyrate activity is mediated by Tregs that stimulate *Wnt10b*. This study connects the microbiota with bone metabolism via T cells.

Interleukin 18 (IL18) is a secreted pro-inflammatory protein expressed by hematopoietic and mesenchymal cells, including osteoblasts. Its receptor is also found on both osteoblasts and osteoclasts. *Il18* expression from osteoblasts is induced *in vitro* with PTH treatment. Therefore, Raggatt et al. studied iPTH in *Il18*^-/-^ mice.^(119)^ The anabolic actions of iPTH were only measured via bone mineral density, which showed no significant differences in the total or cortical bone mineral density response to iPTH between *Il18* knockout and wildtype mice. Trabecular bone mineral density was not significantly increased with iPTH in knockout mice as it was in wildtype mice, although there was a trend. Although IL18 gene and protein expression were significantly upregulated in osteoblasts *in vitro*, data suggests that IL18 is not required for an *in vivo* anabolic response to iPTH; although a more thorough analysis would be warranted to confirm.

PTH stimulates the secretion of interleukin 6 (IL6), which can mediate PTH-dependent hematopoietic cell expansion.^(120)^ Furthermore, IL6 induces osteoclastogenesis and mediates bone resorbing actions of iPTH.^(121)^ Cho et al. used *Il6* knockout and wildtype mice to evaluate the anabolic effect of iPTH.^(122)^ Both genotypes responded normally to iPTH for both growing and adult age models. They hypothesized that the soluble IL6 receptor (sIL6r) was accommodating for the lack of IL6, and designed an experiment to inject *IL6*^-/-^ mice with PTH and soluble glycoprotein 130 receptor (sgp130, blocks sIL6r). Under these conditions, the anabolic response from the iPTH was attenuated in both the femur and vertebrae. This suggests that the IL-6 axis has critical functions in supporting PTH actions in bone.

Milk fat globule-epidermal growth factor 8 (MFGE8) is a pro-resolving glycoprotein involved in apoptotic cell clearance which serves as a bridging molecule between apoptotic and phagocytic cells. It is also a positive regulator of bone turnover, but the exact role in bone is unclear. ^(123)^ Michalski et al. investigated the response of *Mfge8*^-/-^ mice to iPTH. ^(124)^ Baseline bone volume decreased with age in *Mfge8* mutant mice and signs of inflammation increased. After six weeks of iPTH, adult female mice had increased bone volume 1.6-fold in wildtype and a significantly greater 2.3-fold in knockout mice. *Mfge8^-/-^* mice displayed signs of inflammation that iPTH reduced (e.g. number of peripheral neutrophils) suggesting that anabolic iPTH is more robust under certain inflammatory conditions.

Macrophage chemoattractant protein-1 (MCP-1; also known as CCL-2) is highly expressed in bone following treatment with iPTH.^(125)^ It functions to stimulate macrophage and monocyte recruitment by binding to a G-protein coupled receptor, CCR2.^(126)^ When bone is inflamed, osteoblasts are the primary cells that express MCP-1.^(127)^ Tamasi et al. explored iPTH in *Mcp-1*^-/-^ mice, finding that following iPTH treatment, trabecular bone volume was decreased in knockouts compared to the response in wildtype mice (0.084-fold change).^(128)^ Accordingly, the increased osteoclast covered bone surface and osteoclast number seen in wildtype mice treated with iPTH was not observed in *Mcp-1*^-/-^ mice. Data indicates that *Mcp-1* expression is important for the anabolic action of iPTH.

A growing number of studies are examining the effect of macrophages with iPTH. Osteal macrophages are a subset of bone marrow hematopoietic cells that support osteoblast differentiation and mineralization.^(129)^ In order to understand the effects of iPTH in the absence of osteal macrophages, two murine depletion models were used by Cho et al.^(130)^ MAFIA mice were treated with a dimerizing ligand, followed by six weeks of iPTH administration. The second model used clodronate liposomes, which were injected into wildtype mice to deplete phagocytic cells before starting iPTH. The MAFIA mice had a decreased anabolic response to iPTH compared to wildtype, but clodronate treated mice increased their anabolic response compared to PBS liposomes. The clodronate liposomes induced apoptosis in macrophages, which recruited more macrophages and hence changed the bone marrow into an osteogenic environment. The MAFIA model provides a more focused impact of macrophages on anabolic iPTH. Bone volumes were decreased in macrophage depleted MAFIA mice treated with iPTH more than in control mice. Serum P1NP lowered with iPTH in these mice, while TRAP5b showed a mild suppression, indicating that the reduced anabolic response was due to a lack of bone formation. When comparing these models, it is of note that early lineage macrophage depletion (MAFIA mice) and mature macrophage depletion (liposomes) had differing effects with iPTH. Osteal macrophages play an integral role in osteoinductive iPTH that is stage or function dependent and may be attributed to the efferocytic function of macrophages.^(131)^

Extracellular Matrix Proteins

The extracellular matrix (ECM) of bone is comprised of hundreds of proteins that serve as a scaffold for mineralization, direct remodeling, and provide structural flexibility as reviewed by Alford et al. in 2015.^(132)^ Among the ECM proteins is a family of small integrin-binding ligand, N-linked glycoproteins (SIBLINGs), which includes bone sialoprotein (BSP), opteopontin (OPN), dentin matrix protein 1 (DMP1), dentin sialophosphoprotein (DSPP), and matrix extracellular phosphogycoprotein (MEPE). *Bsp* and *Opn* are co-expressed in osteoblasts and osteoclasts.

When *Bsp*^-/-^ mice were injected locally with PTH(1-84) over the calvaria, they had a similar anabolic response as wildtype mice.^(133)^ A thorough analysis of the calvaria included osteogenesis measurements (bone volume, thickness, porosity, mineral density), parietal cellular activity measurements (bone thickness, mineral apposition rate, and bone formation rate), and gene expression markers *in vivo*. Based on this, authors hypothesized that another protein was compensating for the lack of *Bsp* and turned to *Opn*. Using siRNA to block OPN in *BSP*^-/-^ and *BSP*^+/+^ mice, the anabolic effect of iPTH was slightly inhibited in the knockout mice. Taken together, this suggests that these proteins have redundant functions *in vivo* and that they inform in part the anabolic action of iPTH.

OPN, another SIBLING protein that is responsive to PTH, is expressed in both osteoclasts and osteoblasts.^(134)^ *Opn* deficient mice were treated with iPTH for four weeks, and the results showed an increased anabolic response to iPTH compared to wildtype mice.^(135)^­ The bone mineral density for whole femora and tibia were increased with iPTH treatment in *Opn*^-/-^ mice. Bone formation and mineral apposition rates were also enhanced in *Opn*^-/-^ mice. These results show that OPN deficiency increases the anabolic response of PTH.

Periostin (Postn) is a matricellular ECM protein expressed by osteocytes and periosteal osteoblasts that binds to the integrin αvβ3. Authors conclude that *Postn^-/-^* mice have an inhibited response to PTH, based on bone mass, cortical bone volume, and strength response. The focus of this analysis is trabecular bone volume, which was increased in the femur modestly and in the vertebrae (1.106- and 1.762-fold, respectively). In the absence of *Postn*, iPTH does not inhibit *Sost* or induce βcat signaling. Taken together, this paper demonstrates that *Postn* has a complex role in bone anabolism.^(136)^

Osteonectin (ON, also known as secreted protein acidic and rich in cysteine SPARC) is the most abundant non-collagenous protein in bone, with roles in bone mineralization, cell-matrix interactions, and collagen binding. Osteonectin null mice exhibit an osteopenic phenotype, with decreased numbers of surface osteoblasts and osteoclasts and lower BFR. In response to four weeks of iPTH treatment in adult mice, the ratio of osteoblast to osteoclast numbers was decreased in *On*^-/-^ mice compared to *On*^+/+^. Accordingly, the percent of bone surface eroded was higher in heterozygous and knockout mice compared to wildtype. Changes in the anabolic response were attributed to deficient osteoclast formation in the *On^-/-^* model.^(137)^

Proteoglycan 4 (*Prg4*) is a secreted glycoprotein, implicated in protection of articular joints, expansion of hematopoietic progenitor cells, and regulation of megakaryopoiesis. *Prg4* is also endogenously expressed in bone and is a PTH-responsive gene.^(138)^ iPTH treatment of young (four day old) and adult (sixteen-week old) mice showed differing effects, with young mice responding normally to anabolic iPTH. However, adult mice had blunted iPTH-mediated increases in bone mass and femoral bone volume (-9.692-fold change in the anabolic response to PTH in *Prg4^-/-^* mice compared to the iPTH response in wildtype mice). At the cellular level, iPTH responses in *Prg4^-/-^* mice were normal (trabecular bone osteoclast numbers, osteoid surface, osteoclast numbers, BFR, and MAR). Of note, the *Prg4^-/-^* mice had limited joint movement, which may have affected their mechanical loading, a likely co-factor in iPTH actions.^(139)^

The extracellular matrix undergoes normal turnover with degradation by matrix metalloproteinases (MMPs). Collagenase-3, an MMP from osteoblasts is upregulated in response to PTH.^(140)^ In order to understand how MMPs impact iPTH effects on bone, mice that overexpress tissue inhibitor of metalloproteinase 1 (TIMP1) under the promoter for type 1 collagen were used.^(141)^ TIMP1 is a natural inhibitor of metalloproteinases, and this model has been used to show that MMPs from osteoblasts are involved in bone turnover.^(142)^ Administration of iPTH in the transgenic mouse resulted in increased anabolic responses with enhanced bone volume 1.964-fold higher than the wildtype increase. Additionally, the mineral apposition rate increased. A likely explanation for this result is that in response to iPTH, TIMP-1 inhibits bone resorption, as measured by osteoclast number and surface.

Cytoskeletal

Plastin-3 (PLS3) is a member of a protein family that binds to individual actin filaments to promote actin bundle formation.^(143)^ Mutations in *Pls3* are associated with X-linked osteoporosis and fractures.^(144)^ A pilot study in 2017 reported that PLS3 mutation related early onset osteoporosis could be successfully treated with iPTH.^(145)^ Yorgan and colleagues report that there are not differences in the trabecular bone mass or histomorphometry of 12 week old wildtype and *Pls3* deficient mice ^(146)^. However, the tibia and femur of *Pls3* deficient mice showed reduced cortical thickness. The *Pls3* deficient mice were also treated with iPTH for two weeks, and responded similarly to wildtype controls. Relative to untreated mice, there were similar increases in osteoblasts, bone formation rate, and cortical thickness of the femur. This supports treatment of patients with PLS3 mutations, and indicates that there is not a role for PLS3 in the anabolic response to iPTH.

Rac, a subfamily of the Rho GTPases, are small signaling proteins that are involved in structural changes to the actin cytoskeleton. Members Rac1 and Rac2 have been studied with iPTH. Huck and colleagues examined Rac1 in the anabolic response to iPTH ^(147)^. Using a Rac1 inhibitor, osteoblast differentiation decreased. Using *Osx-cre;Rac1^fl/fl^* mice treated with iPTH, authors showed that *Rac1* is not necessary for the anabolic response. Although *Rac1* enhances osteoblast differentiation *in vitro*, there was no effect *in vivo.*

Rac family small GTPase 2 (Rac2) is expressed in hematopoietic cells. Osteoclasts from *Rac2*^-/-^ mice have reduced resorption and migration *in vitro*. Accordingly, the mice have increased trabecular bone mass and osteoclast numbers. iPTH had an enhanced anabolic effect in *Rac2* knockout mice, with bone mineral density increasing in the spine, femur, and total body to a greater extent than in wildtype mice.^(148)^ After iPTH treatment, the serum resorption marker CTX was lower in knockout mice than wildtype mice, although vehicle-treated values were not published to compare changes from PTH. Bone formation markers osteocalcin and P1NP were not different.

Dystrophin is a cytoplasmic protein that connects cytoplasm to the extracellular matrix through a complex known as the dystrophin-associated protein complex. Murine, X-linked mutations in this gene (referred to as *Mdx* mice) result in a phenotype similar to Duchenne muscular dystrophy (DMD). This human disease is marked by progressive muscle strength loss in males between 12-15 years old, and patients are treated with glucocorticoids. This increases the risk for fracture and decreases bone-health markers such as bone mineral content and bone mineral density.^(149)^ *Mdx* and control mice were treated from four to ten weeks of age with 28 nmol/kg/day black bear PTH(1-84) for 5 days per week.^(150)^ Although vehicle treated *Mdx* mice had a lower femoral bone volume than wildtype controls, they had an increased anabolic response to iPTH. Trabecular number and thickness were also increased, although cortical thickness and area was comparable. Since dystrophin deficiency alters calcium signaling, this is a plausible mechanism for affecting anabolic responses to iPTH.

Connexin 43 forms gap junctions, allowing for communication between different cells including osteocytes, osteoblasts, and osteoclasts. The carboxy-terminal allows for opening of the channel and intracellular signaling. Mice with a mutation in the carboxy terminal of one Cx43 allele have lower trabecular bone volume, but increased cortical thickness ^(151)^. With iPTH treatment, *DMP1-Cre;Cx43^ΔCT/fl^* mice respond comparably to controls with increased bone mass and bone formation. Hence reduced *Cx43* in late osteoblasts and osteocytes does not alter anabolic actions yet the full extent of Cx43 in anabolic actions is not known.

Calcium Regulation

The calcium concentration in the bone microenvironment is under tight control, and is detected by the calcium sensing receptor, CaSR. Osteoblasts express CaSR, but its function is not well understood. The CaSR is a G protein coupled receptor that can regulate PTH secretion. Deficiency of CaSR in patients presents with a rickets-like bone phenotype.^(152)^ The role of CaSR in bone was studied in response to iPTH using mice with conditional *CaSR* deletion in osteoblasts, using the 2.3 kb collagen I cre transgene.^(153)^ The anabolic response to iPTH was ablated in mice with conditional knockout of *CaSR* in osteoblasts. There was no significant effect of iPTH on bone volume in knockout mice, even though it was anabolic in wildtype mice. Accordingly, the CaSR is important for the full potential of iPTH.

PTH and active vitamin D (1,25(OH)_2_D) are both regulators of calcium homeostasis. When both PTH and the 25-hydroxyvitamin D-1α-hydroxylase, 1α(OH)ase enzyme (necessary to synthesize 1,25(OH)_2_D) are deleted in mice, there is no calcium-regulating compensatory mechanism.^(154)^ Therefore, 1α(OH)ase knockout mice were maintained on a rescue diet and treated with PTH to understand this interaction.^(155)^ Baseline bone volume, bone formation rates, osteoclast number, serum TRAP, osteoblast number, and serum osteocalcin were lower in the knockout mice, however they were all increased similarly to wildtype mice after iPTH administration. Results indicate that active vitamin D is not necessary for the response of anabolic doses of PTH.

*Summary:* There are data to suggest that there is an effect of apoptosis mechanisms on iPTH, however there are few studies in the literature and further elucidation is needed. The immune system has an intimate relationship with anabolic iPTH. T cells express PTH1R and enhance the anabolic response in bone via Wnt10b production and CD40L interaction with stromal cells. CD8 and T regulatory cells have been specifically identified to be integral for the response in bone to iPTH. There are additional interactions between immunity and anabolic bone metabolism, including via IL6 and phagocytic cell function. For example, loss of MCP-1 results in decreased anabolic response, suggesting that macrophages play a key role in iPTH.

Extracellular matrix proteins have been ablated to study the effect of iPTH. While some models had enhanced iPTH anabolism (*Opn*), others were diminished (*Bsp, Prg4*). Across the board, there are differing results with different ages and treatment regimens. Disruption of cytoskeletal players does not have a significant effect on anabolic iPTH, except for mutations in dystrophin mimicking human disease Duchenne muscular dystrophy. Calcium sensing receptor is required for anabolic iPTH, however active vitamin D is not, indicating a complex mechanism between calcium regulation and iPTH.

***Others***

Cyclin-dependent kinase 1 (Cdk1) is a cell cycle regulator and kinase affecting transcription and development.^(156)^ Knocking out *Cdk1* from osterix cre positive cells resulted in an osteoporotic skeletal phenotype with reduced osteoblast numbers and lower bone formation.^(157)^ However, with iPTH treatment, the conditional knockouts responded with a slightly increased bone volume response vs wildtype controls, including increased osteoblast number, and increased bone formation rates. The number of osteoclasts was unchanged. Overall, osteoblast-specific loss of function suggests that *Cdk1* is important in bone, but disposable in the anabolic response to iPTH.

Hypoxia inducible factors (HIF) control homeostatic responses to low oxygen. In development, Hif-1α can induce angiogenesis for osteoblast specification but in mature bone, Hif-1α is a negative regulator of bone formation.^(158,159)^ In mice lacking *Hif-1α* in osteoblasts and osteocytes, the anabolic response of iPTH reflected by bone volume was enhanced compared to control mice.^(160)^ Since the response to iPTH was not different for serum CTX, but increased for P1NP in mutant mice compared to wildtype, authors suggest that the change in the bone phenotype may be due to increased osteoblast sensitivity to iPTH. This work suggests that Hif-1α suppresses anabolic iPTH.

Osteoblasts express oncostatin M (OSM) in a gp130 dependent manner. OSM is a cytokine that stimulates osteoclast development and bone formation. OSM and PTH both activate RANKL production on the same distal enhancer region.^(161)^ Therefore, Walker et al. investigated how the OSM receptor (OSMR) affects iPTH treatment.^(162)^ With an anabolic treatment, *Osmr*^-/-^ mice experienced catabolic bone changes, with a -0.518-fold change in the bone volume response to iPTH compared to wildtype controls. Trabecular number change was also decreased. Data show that RANKL induction was prolonged in osteoblasts from OSMR deficient mice treated with iPTH compared to wildtype osteoblasts. This indicates that OSMR works to dampen the critical response of RANKL to iPTH.

Hyperlipidemia is more common in patients with osteoporosis. High plasma concentrations of low-density lipoprotein (LDL) is a known risk factor for osteopenia in postmenopausal women.^(163)^ Lipid can deposit in the bone, where osteoblasts can oxidize LDL particles. This can impair bone density *in vivo* and inhibit osteoblast differentiation and enhance osteoclast differentiation *in vitro*. LDL receptor (*Ldlr*) knockout mice are hyperlipidemic, and when treated with iPTH, they have a lower anabolic response than wildtype controls.^(164)^ *Ldlr*^-/-^ mice had a 0.624-fold change in anabolic response when comparing trabecular bone volume. While the mineral apposition rate and bone formation rate increased in knockout mice in response to iPTH, the number of osteoclasts also increased, which likely accounts for the bone volume response.

To better understand the mechanism of iPTH treatment in *Ldlr*^-/-^ mice, they were crossed with mice carrying Col3.6GFPtpz (pre-osteoblasts and osteoblasts are topaz fluorescent) and Col2.3GFPcyan (mature osteoblasts and osteocytes are cyan fluorescent).^(165)^ After treatment with iPTH, trabecular fluorescence intensity was increased for topaz in wildtype, but not *Ldlr^-/-^* mice. Furthermore, the expression of *Pth1r* was reduced, which may account for the dampened response.

The β_2_-adrenergic receptor (β_2_AR) is a G protein coupled receptor that activates the cAMP/PKA signal transduction pathway, a common pathway stimulated via PTH1R ligand binding. However, activation of β_2_AR on osteoblasts decreases bone formation and increases bone resorption.^(166)^ In mice lacking the *β_2_AR*, iPTH had no anabolic activity.^(167)^ This was shown by μCT analysis, mineral apposition rate, bone formation rate, mineralizing surface, and osteoclast numbers. There was also decreased expression of PTH reactive genes (alkaline phosphatase, type 1 collagen α1, Runx2, osterix) in *β_2_AR*^-/-^ mice relative to the iPTH induced increase in wildtype mice. These results were noted in the femur and vertebrae of adult (ten weeks) and aged mice (54 weeks). Given the previous reports of β_2_AR exerting negative effects on bone mass, it is surprising that *β_2_AR* knockout mice lack an anabolic response to iPTH, however osteoblasts require this receptor for proper signaling. Since both β_2_AR and PTH1R activate cAMP/PKA signal transduction, it is possible that these distinct receptors converge for the full anabolic response.

Patients with Trisomy 21 clinically present with Down syndrome and reported low bone mineral density.^(168)^ The effects of trisomy 21 on the skeleton have not been well studied. With a murine model for Down syndrome, Ts65Dn mice, Fowler et al. studied the effect of iPTH.^(169)^ Mineral apposition rate and bone formation rate were increased to a similar extent in Ts65Dn and wildtype mice with iPTH, despite a low baseline bone mineral density in Ts65Dn mice without treatment. This data supports potential efficacy of teriparatide in patients with trisomy 21 with osteoporosis.

Cyclooxygenase (COX) regulates prostaglandins, which are multifunctional regulators that can stimulate both bone resorption and formation.^(170)^ COX2 is induced in osteoblasts by PTH, and is responsible for most prostaglandin production in bone. *Cox2*^-/-^ mice were administered iPTH, and anabolic actions were increased similarly to that of wildtype treated mice.^(171)^ Femoral bone volume was increased, and the number of osteoblasts, osteoclasts, and bone formation rates all responded similarly. However, the ratio of osteoblasts to osteoclasts was increased in treated knockout mice. It is not clear from this study that downstream prostaglandins are not involved in anabolic iPTH because COX1, an isoform of COX, may have compensatory mechanisms.

Small, single-stranded, non-coding microRNAs can post-transcriptionally fine-tune gene expression, including regulation in osteoclasts and osteoblasts. The miRNA family miRNA-29-3p is particularly involved in osteoclast and osteoblast differentiation and function.^(172)^ Hrdlicka and colleagues created a mouse model with a globally expressed miR-29-3p tough decoy, which is a competitive inhibitor to study the effect of miR-29-3p activity.^(173)^ The miR-29-3p tough decoy mice have decreased trabecular bone volume. When treated with iPTH for four weeks, the anabolic response in the decoy mice is enhanced, as measured by an approximate 8.858 fold increase in the bone volume of femurs, increased bone formation rate, and increased osteoblasts. MicroRNA inhibitors may be a useful co-therapeutic with iPTH in the future, as these results support a role for miR-29-3p in dampening the anabolic responses.

Overall, the anabolic actions of iPTH are broadly affected via multiple and diverse genes. This paper details decades of work to highlight the importance of many cell types and expression patterns in the bone microenvironment.

*Summary:* Of note are different mutations that affect anabolic iPTH. This includes *Hif-1α*, a transcription factor used in response to low oxygen, which reduces the anabolic response. OSMR dampens the response of RANKL to iPTH, therefore plays a role in enhancing the anabolic mechanism. Proper lipid metabolism has also been studied in the context of iPTH, and the LDL receptor plays a critical role in iPTH. While the PTH1R receptor works through the cAMP signal transduction pathway, so do other receptors, including β_2_AR. This overlap may account for the lack of an anabolic response to iPTH when β_2_AR is ablated. MicroRNA regulation may be a potential therapeutic way to fine tune bone anabolic metabolism, as they post-transcriptionally affect gene expression and inhibition of microRNA miR-29-3p enhances anabolic iPTH.

*Overall Summary:* There are a large number of genes that can influence skeletal health and significant dimorphism between the sexes and bone compartments adds to the complexity of the skeletal findings.^(174)^ Analyzing the PTH effects on top of the skeletal complexity adds another dimension, yet with twenty years of studies the importance of many genes and the relative lack of importance of others is becoming clearer. When mice are administered anabolic doses of PTH, signaling cascades affect proliferation and development of osteoblasts. There are many protein interactions and regulatory factors involved in this process, and it is unsurprising that when they are disrupted, the anabolic response is affected. The purpose of this study was to further elucidate PTH mechanisms by collectively analyzing the extensive work performed using mouse models.

**References**

1. Miao D, He B, Jiang Y, Kobayashi T, Soroceanu MA, Zhao J, et al. Osteoblast-derived PTHrP is a potent endogenous bone anabolic agent that modifies the therapeutic efficacy of administered PTH 1-34. J Clin Invest. Sep 2005;115(9):2402-11.

2. Delgado-Calle J, Tu X, Pacheco-Costa R, McAndrews K, Edwards R, Pellegrini GG, et al. Control of bone anabolism in response to mechanical loading and PTH by distinct mechanisms downstream of the PTH receptor. J Bone Miner Res. Mar 2017;32(3):522-35.

3. Saini V, Marengi DA, Barry KJ, Fulzele KS, Heiden E, Liu X, et al. Parathyroid hormone (PTH)/PTH-related peptide type 1 receptor (PPR) signaling in osteocytes regulates anabolic and catabolic skeletal responses to PTH. J Biol Chem. Jul 12 2013;288(28):20122-34.

4. Sinha P, Aarnisalo P, Chubb R, Poulton IJ, Guo J, Nachtrab G, et al. Loss of Gsα in the Postnatal Skeleton Leads to Low Bone Mass and a Blunted Response to Anabolic Parathyroid Hormone Therapy. J Biol Chem. Jan 22 2016;291(4):1631-42.

5. Schipani E, Kruse K, Juppner H. A constitutively active mutant PTH-PTHrP receptor in Jansen-type metaphyseal chondrodysplasia. Science. Apr 7 1995;268(5207):98-100.

6. Sneddon WB, Magyar CE, Willick GE, Syme CA, Galbiati F, Bisello A, et al. Ligand-selective dissociation of activation and internalization of the parathyroid hormone (PTH) receptor: conditional efficacy of PTH peptide fragments. Endocrinology. Jun 2004;145(6):2815-23.

7. Wang B, Yang Y, Abou-Samra AB, Friedman PA. NHERF1 regulates parathyroid hormone receptor desensitization: interference with β-arrestin binding. Mol Pharmacol. May 2009;75(5):1189-97.

8. Datta NS, Samra TA, Abou-Samra AB. Parathyroid hormone induces bone formation in phosphorylation-deficient PTHR1 knockin mice. Am J Physiol Endocrinol Metab. May 1 2012;302(10):E1183-8.

9. Spurney RF, Flannery PJ, Garner SC, Athirakul K, Liu S, Guilak F, et al. Anabolic effects of a G protein-coupled receptor kinase inhibitor expressed in osteoblasts. J Clin Invest. May 2002;109(10):1361-71.

10. Wang L, Quarles LD, Spurney RF. Unmasking the osteoinductive effects of a G-protein-coupled receptor (GPCR) kinase (GRK) inhibitor by treatment with PTH(1-34). J Bone Miner Res. Oct 2004;19(10):1661-70.

11. Luo J, Sun P, Siwko S, Liu M, Xiao J. The role of GPCRs in bone diseases and dysfunctions. Bone Res. 2019;7:19.

12. van der Valk RJ, Kreiner-Moller E, Kooijman MN, Guxens M, Stergiakouli E, Saaf A, et al. A novel common variant in DCST2 is associated with length in early life and height in adulthood. Hum Mol Genet. Feb 15 2015;24(4):1155-68.

13. Sun P, He L, Jia K, Yue Z, Li S, Jin Y, et al. Regulation of body length and bone mass by Gpr126/Adgrg6. Sci Adv. Mar 2020;6(12):eaaz0368.

14. McGough IJ, Cullen PJ. Recent advances in retromer biology. Traffic. Aug 2011;12(8):963-71.

15. Xia WF, Tang FL, Xiong L, Xiong S, Jung JU, Lee DH, et al. Vps35 loss promotes hyperresorptive osteoclastogenesis and osteoporosis via sustained RANKL signaling. J Cell Biol. Mar 18 2013;200(6):821-37.

16. Xiong L, Xia WF, Tang FL, Pan JX, Mei L, Xiong WC. Retromer in osteoblasts interacts with protein phosphatase 1 regulator subunit 14C, terminates parathyroid hormone's signaling, and promotes its catabolic response. EBioMedicine. Jul 2016;9:45-60.

17. Nervina JM, Tetradis S, Huang YF, Harrison D, Molina C, Kream BE. Expression of inducible cAMP early repressor is coupled to the cAMP-protein kinase A signaling pathway in osteoblasts. Bone. May 2003;32(5):483-90.

18. Liu F, Lee SK, Adams DJ, Gronowicz GA, Kream BE. CREM deficiency in mice alters the response of bone to intermittent parathyroid hormone treatment. Bone. Apr 2007;40(4):1135-43.

19. Saito H, Gasser A, Bolamperti S, Maeda M, Matthies L, Jahn K, et al. TG-interacting factor 1 (Tgif1)-deficiency attenuates bone remodeling and blunts the anabolic response to parathyroid hormone. Nat Commun. Mar 22 2019;10(1):1354.

20. Yu S, Franceschi RT, Luo M, Fan J, Jiang D, Cao H, et al. Critical role of activating transcription factor 4 in the anabolic actions of parathyroid hormone in bone. PLoS One. Oct 23 2009;4(10):e7583.

21. Koh AJ, Demiralp B, Neiva KG, Hooten J, Nohutcu RM, Shim H, et al. Cells of the osteoclast lineage as mediators of the anabolic actions of parathyroid hormone in bone. Endocrinology. Nov 2005;146(11):4584-96.

22. Demiralp B, Chen HL, Koh AJ, Keller ET, McCauley LK. Anabolic actions of parathyroid hormone during bone growth are dependent on c-fos. Endocrinology. Oct 2002;143(10):4038-47.

23. Koh AJ, Novince CM, Li X, Wang T, Taichman RS, McCauley LK. An irradiation-altered bone marrow microenvironment impacts anabolic actions of PTH. Endocrinology. Dec 2011;152(12):4525-36.

24. Shah R, Alvarez M, Jones DR, Torrungruang K, Watt AJ, Selvamurugan N, et al. Nmp4/CIZ regulation of matrix metalloproteinase 13 (MMP-13) response to parathyroid hormone in osteoblasts. Am J Physiol Endocrinol Metab. Aug 2004;287(2):E289-96.

25. Shen ZJ, Nakamoto T, Tsuji K, Nifuji A, Miyazono K, Komori T, et al. Negative regulation of bone morphogenetic protein/Smad signaling by Cas-interacting zinc finger protein in osteoblasts. J Biol Chem. Aug 16 2002;277(33):29840-6.

26. Robling AG, Childress P, Yu J, Cotte J, Heller A, Philip BK, et al. Nmp4/CIZ suppresses parathyroid hormone-induced increases in trabecular bone. J Cell Physiol. Jun 2009;219(3):734-43.

27. Childress P, Philip BK, Robling AG, Bruzzaniti A, Kacena MA, Bivi N, et al. Nmp4/CIZ suppresses the response of bone to anabolic parathyroid hormone by regulating both osteoblasts and osteoclasts. Calcif Tissue Int. Jul 2011;89(1):74-89.

28. He Y, Childress P, Hood M, Jr., Alvarez M, Kacena MA, Hanlon M, et al. Nmp4/CIZ suppresses the parathyroid hormone anabolic window by restricting mesenchymal stem cell and osteoprogenitor frequency. Stem Cells Dev. Feb 1 2013;22(3):492-500.

29. Ferrari SL, Pierroz DD, Glatt V, Goddard DS, Bianchi EN, Lin FT, et al. Bone response to intermittent parathyroid hormone is altered in mice null for β-Arrestin2. Endocrinology. Apr 2005;146(4):1854-62.

30. Bisello A, Chorev M, Rosenblatt M, Monticelli L, Mierke DF, Ferrari SL. Selective ligand-induced stabilization of active and desensitized parathyroid hormone type 1 receptor conformations. J Biol Chem. Oct 11 2002;277(41):38524-30.

31. Gesty-Palmer D, Flannery P, Yuan L, Corsino L, Spurney R, Lefkowitz RJ, et al. A β-arrestin-biased agonist of the parathyroid hormone receptor (PTH1R) promotes bone formation independent of G protein activation. Sci Transl Med. Oct 7 2009;1(1):1ra.

32. Bianchi EN, Ferrari SL. β-arrestin2 regulates parathyroid hormone effects on a p38 MAPK and NFkappaB gene expression network in osteoblasts. Bone. Oct 2009;45(4):716-25.

33. Thouverey C, Caverzasio J. Suppression of p38α MAPK signaling in osteoblast lineage cells impairs bone anabolic action of parathyroid hormone. J Bone Miner Res. May 2016;31(5):985-93.

34. Oakhill JS, Scott JW, Kemp BE. Structure and function of AMP-activated protein kinase. Acta Physiol (Oxf). May 2009;196(1):3-14.

35. Shah M, Kola B, Bataveljic A, Arnett TR, Viollet B, Saxon L, et al. AMP-activated protein kinase (AMPK) activation regulates in vitro bone formation and bone mass. Bone. Aug 2010;47(2):309-19.

36. Jeyabalan J, Shah M, Viollet B, Roux JP, Chavassieux P, Korbonits M, et al. Mice lacking AMP-activated protein kinase α1 catalytic subunit have increased bone remodelling and modified skeletal responses to hormonal challenges induced by ovariectomy and intermittent PTH treatment. J Endocrinol. Sep 2012;214(3):349-58.

37. Chen C, Koh AJ, Datta NS, Zhang J, Keller ET, Xiao G, et al. Impact of the mitogen-activated protein kinase pathway on parathyroid hormone-related protein actions in osteoblasts. J Biol Chem. Jul 9 2004;279(28):29121-9.

38. Xiao G, Jiang D, Gopalakrishnan R, Franceschi RT. Fibroblast growth factor 2 induction of the osteocalcin gene requires MAPK activity and phosphorylation of the osteoblast transcription factor, Cbfa1/Runx2. J Biol Chem. Sep 27 2002;277(39):36181-7.

39. Hurley MM, Marcello K, Abreu C, Kessler M. Signal transduction by basic fibroblast growth factor in rat osteoblastic Py1a cells. J Bone Miner Res. Sep 1996;11(9):1256-63.

40. Ge C, Xiao G, Jiang D, Franceschi RT. Critical role of the extracellular signal-regulated kinase-MAPK pathway in osteoblast differentiation and skeletal development. J Cell Biol. Feb 26 2007;176(5):709-18.

41. Mahalingam CD, Datta T, Patil RV, Kreider J, Bonfil RD, Kirkwood KL, et al. Mitogen-activated protein kinase phosphatase 1 regulates bone mass, osteoblast gene expression, and responsiveness to parathyroid hormone. J Endocrinol. Nov 2011;211(2):145-56.

42. Yu X, Chen S, Potter OL, Murthy SM, Li J, Pulcini JM, et al. Neurofibromin and its inactivation of Ras are prerequisites for osteoblast functioning. Bone. May 2005;36(5):793-802.

43. Yu X, Milas J, Watanabe N, Rao N, Murthy S, Potter OL, et al. Neurofibromatosis type 1 gene haploinsufficiency reduces AP-1 gene expression without abrogating the anabolic effect of parathyroid hormone. Calcif Tissue Int. Mar 2006;78(3):162-70.

44. Ducy P, Starbuck M, Priemel M, Shen J, Pinero G, Geoffroy V, et al. A Cbfa1-dependent genetic pathway controls bone formation beyond embryonic development. Genes Dev. Apr 15 1999;13(8):1025-36.

45. Komori T, Yagi H, Nomura S, Yamaguchi A, Sasaki K, Deguchi K, et al. Targeted disruption of Cbfa1 results in a complete lack of bone formation owing to maturational arrest of osteoblasts. Cell. May 30 1997;89(5):755-64.

46. Liu W, Toyosawa S, Furuichi T, Kanatani N, Yoshida C, Liu Y, et al. Overexpression of Cbfa1 in osteoblasts inhibits osteoblast maturation and causes osteopenia with multiple fractures. J Cell Biol. Oct 1 2001;155(1):157-66.

47. Merciris D, Marty C, Collet C, de Vernejoul MC, Geoffroy V. Overexpression of the transcriptional factor Runx2 in osteoblasts abolishes the anabolic effect of parathyroid hormone in vivo. Am J Pathol. May 2007;170(5):1676-85.

48. McCauley LK, Koh AJ, Beecher CA, Cui Y, Decker JD, Franceschi RT. Effects of differentiation and transforming growth factor β 1 on PTH/PTHrP receptor mRNA levels in MC3T3-E1 cells. J Bone Miner Res. Aug 1995;10(8):1243-55.

49. Montero A, Okada Y, Tomita M, Ito M, Tsurukami H, Nakamura T, et al. Disruption of the fibroblast growth factor-2 gene results in decreased bone mass and bone formation. J Clin Invest. Apr 2000;105(8):1085-93.

50. Hurley MM, Tetradis S, Huang YF, Hock J, Kream BE, Raisz LG, et al. Parathyroid hormone regulates the expression of fibroblast growth factor-2 mRNA and fibroblast growth factor receptor mRNA in osteoblastic cells. J Bone Miner Res. May 1999;14(5):776-83.

51. Zhang X, Sobue T, Hurley MM. FGF-2 increases colony formation, PTH receptor, and IGF-1 mRNA in mouse marrow stromal cells. Biochem Biophys Res Commun. Jan 11 2002;290(1):526-31.

52. Hurley MM, Okada Y, Xiao L, Tanaka Y, Ito M, Okimoto N, et al. Impaired bone anabolic response to parathyroid hormone in Fgf2-/- and Fgf2+/- mice. Biochem Biophys Res Commun. Mar 24 2006;341(4):989-94.

53. Hurley M, Yao W, Lane NE. Changes in serum fibroblast growth factor 2 in patients with glucocorticoid-induced osteoporosis treated with human parathyroid hormone (1-34). Osteoporos Int. Dec 2005;16(12):2080-4.

54. Sabbieti MG, Agas D, Xiao L, Marchetti L, Coffin JD, Doetschman T, et al. Endogenous FGF-2 is critically important in PTH anabolic effects on bone. J Cell Physiol. Apr 2009;219(1):143-51.

55. Fei Y, Xiao L, Hurley MM. The impaired bone anabolic effect of PTH in the absence of endogenous FGF2 is partially due to reduced ATF4 expression. Biochem Biophys Res Commun. Aug 19 2011;412(1):160-4.

56. Xiao L, Fei Y, Hurley MM. FGF2 crosstalk with Wnt signaling in mediating the anabolic action of PTH on bone formation. Bone Rep. Dec 2018;9:136-44.

57. Valverde-Franco G, Liu H, Davidson D, Chai S, Valderrama-Carvajal H, Goltzman D, et al. Defective bone mineralization and osteopenia in young adult FGFR3-/- mice. Hum Mol Genet. Feb 1 2004;13(3):271-84.

58. Xie Y, Yi L, Weng T, Huang J, Luo F, Jiang W, et al. Fibroblast growth factor receptor 3 deficiency does not impair the osteoanabolic action of parathyroid hormone on mice. Int J Biol Sci. 2016;12(8):990-9.

59. Chen L, Adar R, Yang X, Monsonego EO, Li C, Hauschka PV, et al. Gly369Cys mutation in mouse FGFR3 causes achondroplasia by affecting both chondrogenesis and osteogenesis. J Clin Invest. Dec 1999;104(11):1517-25.

60. Chen H, Sun X, Yin L, Chen S, Zhu Y, Huang J, et al. PTH 1-34 ameliorates the osteopenia and delayed healing of stabilized tibia fracture in mice with achondroplasia resulting from gain-of-function mutation of FGFR3. Int J Biol Sci. 2017;13(10):1254-65.

61. Kawata T, Imanishi Y, Kobayashi K, Miki T, Arnold A, Inaba M, et al. Parathyroid hormone regulates fibroblast growth factor-23 in a mouse model of primary hyperparathyroidism. J Am Soc Nephrol. Oct 2007;18(10):2683-8.

62. Yuan Q, Sato T, Densmore M, Saito H, Schuler C, Erben RG, et al. FGF-23/Klotho signaling is not essential for the phosphaturic and anabolic functions of PTH. J Bone Miner Res. Sep 2011;26(9):2026-35.

63. Rudnicki MA, Williams BO. Wnt signaling in bone and muscle. Bone. Nov 2015;80:60-6.

64. Keupp K, Beleggia F, Kayserili H, Barnes AM, Steiner M, Semler O, et al. Mutations in WNT1 cause different forms of bone fragility. Am J Hum Genet. Apr 4 2013;92(4):565-74.

65. Yorgan TA, Rolvien T, Sturznickel J, Vollersen N, Lange F, Zhao W, et al. Mice carrying a ubiquitous R235W mutation of Wnt1 display a bone-specific phenotype. J Bone Miner Res. Sep 2020;35(9):1726-37.

66. Sawakami K, Robling AG, Ai M, Pitner ND, Liu D, Warden SJ, et al. The Wnt co-receptor LRP5 is essential for skeletal mechanotransduction but not for the anabolic bone response to parathyroid hormone treatment. J Biol Chem. Aug 18 2006;281(33):23698-711.

67. Iwaniec UT, Wronski TJ, Liu J, Rivera MF, Arzaga RR, Hansen G, et al. PTH stimulates bone formation in mice deficient in Lrp5. J Bone Miner Res. Mar 2007;22(3):394-402.

68. Li C, Xing Q, Yu B, Xie H, Wang W, Shi C, et al. Disruption of LRP6 in osteoblasts blunts the bone anabolic activity of PTH. J Bone Miner Res. Oct 2013;28(10):2094-108.

69. Li C, Wang W, Xie L, Luo X, Cao X, Wan M. Lipoprotein receptor-related protein 6 is required for parathyroid hormone-induced Sost suppression. Ann N Y Acad Sci. Jan 2016;1364:62-73.

70. Revollo L, Kading J, Jeong SY, Li J, Salazar V, Mbalaviele G, et al. N-cadherin restrains PTH activation of Lrp6/ β-catenin signaling and osteoanabolic action. J Bone Miner Res. Feb 2015;30(2):274-85.

71. Kedlaya R, Kang KS, Hong JM, Bettagere V, Lim KE, Horan D, et al. Adult-onset deletion of β-catenin in (10kb) Dmp1-expressing cells prevents intermittent PTH-induced bone gain. Endocrinology. Aug 2016;157(8):3047-57.

72. Yu C, Xuan M, Zhang M, Yao Q, Zhang K, Zhang X, et al. Postnatal deletion of β-catenin in osterix-expressing cells is necessary for bone growth and intermittent PTH-induced bone gain. J Bone Miner Metab. Sep 2018;36(5):560-72.

73. Bodine PV, Zhao W, Kharode YP, Bex FJ, Lambert AJ, Goad MB, et al. The Wnt antagonist secreted frizzled-related protein-1 is a negative regulator of trabecular bone formation in adult mice. Mol Endocrinol. May 2004;18(5):1222-37.

74. Bodine PV, Seestaller-Wehr L, Kharode YP, Bex FJ, Komm BS. Bone anabolic effects of parathyroid hormone are blunted by deletion of the Wnt antagonist secreted frizzled-related protein-1. J Cell Physiol. Feb 2007;210(2):352-7.

75. Yao W, Cheng Z, Shahnazari M, Dai W, Johnson ML, Lane NE. Overexpression of secreted frizzled-related protein 1 inhibits bone formation and attenuates parathyroid hormone bone anabolic effects. J Bone Miner Res. Feb 2010;25(2):190-9.

76. Yao GQ, Wu JJ, Troiano N, Insogna K. Targeted overexpression of Dkk1 in osteoblasts reduces bone mass but does not impair the anabolic response to intermittent PTH treatment in mice. J Bone Miner Metab. Mar 2011;29(2):141-8.

77. Bellido T, Ali AA, Gubrij I, Plotkin LI, Fu Q, O'Brien CA, et al. Chronic elevation of parathyroid hormone in mice reduces expression of sclerostin by osteocytes: a novel mechanism for hormonal control of osteoblastogenesis. Endocrinology. Nov 2005;146(11):4577-83.

78. Kramer I, Loots GG, Studer A, Keller H, Kneissel M. Parathyroid hormone (PTH)-induced bone gain is blunted in SOST overexpressing and deficient mice. J Bone Miner Res. Feb 2010;25(2):178-89.

79. Robling AG, Kedlaya R, Ellis SN, Childress PJ, Bidwell JP, Bellido T, et al. Anabolic and catabolic regimens of human parathyroid hormone 1-34 elicit bone- and envelope-specific attenuation of skeletal effects in Sost-deficient mice. Endocrinology. Aug 2011;152(8):2963-75.

80. Wein MN, Spatz J, Nishimori S, Doench J, Root D, Babij P, et al. HDAC5 controls MEF2C-driven sclerostin expression in osteocytes. J Bone Miner Res. Mar 2015;30(3):400-11.

81. Wein MN, Liang Y, Goransson O, Sundberg TB, Wang J, Williams EA, et al. SIKs control osteocyte responses to parathyroid hormone. Nat Commun. Oct 19 2016;7:13176.

82. Wheelock MJ, Johnson KR. Cadherins as modulators of cellular phenotype. Annu Rev Cell Dev Biol. 2003;19:207-35.

83. Yang H, Dong J, Xiong W, Fang Z, Guan H, Li F. N-cadherin restrains PTH repressive effects on sclerostin/SOST by regulating LRP6-PTH1R interaction. Ann N Y Acad Sci. Dec 2016;1385(1):41-52.

84. Khan MP, Khan K, Yadav PS, Singh AK, Nag A, Prasahar P, et al. BMP signaling is required for adult skeletal homeostasis and mediates bone anabolic action of parathyroid hormone. Bone. Nov 2016;92:132-44.

85. Miyakoshi N, Kasukawa Y, Linkhart TA, Baylink DJ, Mohan S. Evidence that anabolic effects of PTH on bone require IGF-I in growing mice. Endocrinology. Oct 2001;142(10):4349-56.

86. Bikle DD, Sakata T, Leary C, Elalieh H, Ginzinger D, Rosen CJ, et al. Insulin-like growth factor I is required for the anabolic actions of parathyroid hormone on mouse bone. J Bone Miner Res. Sep 2002;17(9):1570-8.

87. Rosen CJ, Ackert-Bicknell C, Beamer WG, Nelson T, Adamo M, Cohen P, et al. Allelic differences in a quantitative trait locus affecting insulin-like growth factor-I impact skeletal acquisition and body composition. Pediatr Nephrol. Mar 2005;20(3):255-60. E

88. Elis S, Courtland HW, Wu Y, Fritton JC, Sun H, Rosen CJ, et al. Elevated serum IGF-1 levels synergize PTH action on the skeleton only when the tissue IGF-1 axis is intact. J Bone Miner Res. Sep 2010;25(9):2051-8.

89. Yakar S, Bouxsein ML, Canalis E, Sun H, Glatt V, Gundberg C, et al. The ternary IGF complex influences postnatal bone acquisition and the skeletal response to intermittent parathyroid hormone. J Endocrinol. May 2006;189(2):289-99.

90. Clifton KB, Conover CA. Pregnancy-associated plasma protein-A modulates the anabolic effects of parathyroid hormone in mouse bone. Bone. Dec 2015;81:413-6.

91. Wang Y, Menendez A, Fong C, ElAlieh HZ, Chang W, Bikle DD. Ephrin B2/EphB4 mediates the actions of IGF-I signaling in regulating endochondral bone formation. J Bone Miner Res. Aug 2014;29(8):1900-13.

92. Wang Y, Nishida S, Boudignon BM, Burghardt A, Elalieh HZ, Hamilton MM, et al. IGF-I receptor is required for the anabolic actions of parathyroid hormone on bone. J Bone Miner Res. Sep 2007;22(9):1329-37.

93. Takyar FM, Tonna S, Ho PW, Crimeen-Irwin B, Baker EK, Martin TJ, et al. EphrinB2/EphB4 inhibition in the osteoblast lineage modifies the anabolic response to parathyroid hormone. J Bone Miner Res. Apr 2013;28(4):912-25.

94. Ogata N, Chikazu D, Kubota N, Terauchi Y, Tobe K, Azuma Y, et al. Insulin receptor substrate-1 in osteoblast is indispensable for maintaining bone turnover. J Clin Invest. Apr 2000;105(7):935-43.

95. Akune T, Ogata N, Hoshi K, Kubota N, Terauchi Y, Tobe K, et al. Insulin receptor substrate-2 maintains predominance of anabolic function over catabolic function of osteoblasts. J Cell Biol. Oct 14 2002;159(1):147-56.

96. Yamaguchi M, Ogata N, Shinoda Y, Akune T, Kamekura S, Terauchi Y, et al. Insulin receptor substrate-1 is required for bone anabolic function of parathyroid hormone in mice. Endocrinology. Jun 2005;146(6):2620-8.

97. Perrini S, Laviola L, Carreira MC, Cignarelli A, Natalicchio A, Giorgino F. The GH/IGF1 axis and signaling pathways in the muscle and bone: mechanisms underlying age-related skeletal muscle wasting and osteoporosis. J Endocrinol. Jun 2010;205(3):201-10.

98. Liu Z, Kennedy OD, Cardoso L, Basta-Pljakic J, Partridge NC, Schaffler MB, et al. DMP-1-mediated Ghr gene recombination compromises skeletal development and impairs skeletal response to intermittent PTH. FASEB J. Feb 2016;30(2):635-52.

99. Schneider MR, Sibilia M, Erben RG. The EGFR network in bone biology and pathology. Trends Endocrinol Metab. Dec 2009;20(10):517-24.

100. Schneider MR, Dahlhoff M, Andrukhova O, Grill J, Glosmann M, Schuler C, et al. Normal epidermal growth factor receptor signaling is dispensable for bone anabolic effects of parathyroid hormone. Bone. Jan 2012;50(1):237-44.

101. Qin L, Qiu P, Wang L, Li X, Swarthout JT, Soteropoulos P, et al. Gene expression profiles and transcription factors involved in parathyroid hormone signaling in osteoblasts revealed by microarray and bioinformatics. J Biol Chem. May 30 2003;278(22):19723-31.

102. Qin L, Tamasi J, Raggatt L, Li X, Feyen JH, Lee DC, et al. Amphiregulin is a novel growth factor involved in normal bone development and in the cellular response to parathyroid hormone stimulation. J Biol Chem. Feb 4 2005;280(5):3974-81.

103. Zhu J, Siclari VA, Liu F, Spatz JM, Chandra A, Divieti Pajevic P, et al. Amphiregulin-EGFR signaling mediates the migration of bone marrow mesenchymal progenitors toward PTH-stimulated osteoblasts and osteocytes. PLoS One. 2012;7(12):e50099.

104. Jay FF, Vaidya M, Porada SM, Andrukhova O, Schneider MR, Erben RG. Amphiregulin lacks an essential role for the bone anabolic action of parathyroid hormone. Mol Cell Endocrinol. Dec 5 2015;417:158-65.

105. Manolagas SC. Birth and death of bone cells: basic regulatory mechanisms and implications for the pathogenesis and treatment of osteoporosis. Endocr Rev. Apr 2000;21(2):115-37.

106. Veis DJ, Sorenson CM, Shutter JR, Korsmeyer SJ. Bcl-2-deficient mice demonstrate fulminant lymphoid apoptosis, polycystic kidneys, and hypopigmented hair. Cell. Oct 22 1993;75(2):229-40.

107. Yamashita J, Datta NS, Chun YH, Yang DY, Carey AA, Kreider JM, et al. Role of Bcl2 in osteoclastogenesis and PTH anabolic actions in bone. J Bone Miner Res. May 2008;23(5):621-32.

108. Nagase Y, Iwasawa M, Akiyama T, Ogata N, Kadono Y, Nakamura M, et al. Antiapoptotic molecule Bcl-2 is essential for the anabolic activity of parathyroid hormone in bone. Ann N Y Acad Sci. Mar 2010;1192:330-7.

109. Zhou Z, Immel D, Xi CX, Bierhaus A, Feng X, Mei L, et al. Regulation of osteoclast function and bone mass by RAGE. J Exp Med. Apr 17 2006;203(4):1067-80.

110. Philip BK, Childress PJ, Robling AG, Heller A, Nawroth PP, Bierhaus A, et al. RAGE supports parathyroid hormone-induced gains in femoral trabecular bone. Am J Physiol Endocrinol Metab. Mar 2010;298(3):E714-25.

111. Rifas L, Arackal S, Weitzmann MN. Inflammatory T cells rapidly induce differentiation of human bone marrow stromal cells into mature osteoblasts. J Cell Biochem. Mar 1 2003;88(4):650-9.

112. Bedi B, Li JY, Tawfeek H, Baek KH, Adams J, Vangara SS, et al. Silencing of parathyroid hormone (PTH) receptor 1 in T cells blunts the bone anabolic activity of PTH. Proc Natl Acad Sci U S A. Mar 20 2012;109(12):E725-33.

113. Terauchi M, Li JY, Bedi B, Baek KH, Tawfeek H, Galley S, et al. T lymphocytes amplify the anabolic activity of parathyroid hormone through Wnt10b signaling. Cell Metab. Sep 2009;10(3):229-40.

114. Robinson JW, Li JY, Walker LD, Tyagi AM, Reott MA, Yu M, et al. T cell-expressed CD40L potentiates the bone anabolic activity of intermittent PTH treatment. J Bone Miner Res. Apr 2015;30(4):695-705.

115. Lopez-Granados E, Temmerman ST, Wu L, Reynolds JC, Follmann D, Liu S, et al. Osteopenia in X-linked hyper-IgM syndrome reveals a regulatory role for CD40 ligand in osteoclastogenesis. Proc Natl Acad Sci U S A. Mar 20 2007;104(12):5056-61.

116. Yuan FL, Li X, Lu WG, Xu RS, Zhao YQ, Li CW, et al. Regulatory T cells as a potent target for controlling bone loss. Biochem Biophys Res Commun. Nov 12 2010;402(2):173-6.

117. Yu M, D'Amelio P, Tyagi AM, Vaccaro C, Li JY, Hsu E, et al. Regulatory T cells are expanded by Teriparatide treatment in humans and mediate intermittent PTH-induced bone anabolism in mice. EMBO Rep. Jan 2018;19(1):156-71.

118. Li JY, Yu M, Pal S, Tyagi AM, Dar H, Adams J, et al. Parathyroid hormone-dependent bone formation requires butyrate production by intestinal microbiota. J Clin Invest. Apr 1 2020;130(4):1767-81.

119. Raggatt LJ, Qin L, Tamasi J, Jefcoat SC, Jr., Shimizu E, Selvamurugan N, et al. Interleukin-18 is regulated by parathyroid hormone and is required for its bone anabolic actions. J Biol Chem. Mar 14 2008;283(11):6790-8.

120. Pirih FQ, Michalski MN, Cho SW, Koh AJ, Berry JE, Ghaname E, et al. Parathyroid hormone mediates hematopoietic cell expansion through interleukin-6. PLoS One. Oct 27 2010;5(10):e13657.

121. Grey A, Mitnick MA, Masiukiewicz U, Sun BH, Rudikoff S, Jilka RL, et al. A role for interleukin-6 in parathyroid hormone-induced bone resorption in vivo. Endocrinology. Oct 1999;140(10):4683-90.

122. Cho SW, Pirih FQ, Koh AJ, Michalski M, Eber MR, Ritchie K, et al. The soluble interleukin-6 receptor is a mediator of hematopoietic and skeletal actions of parathyroid hormone. J Biol Chem. Mar 8 2013;288(10):6814-25.

123. Sinningen K, Albus E, Thiele S, Grossklaus S, Kurth T, Udey MC, et al. Loss of milk fat globule-epidermal growth factor 8 (MFG-E8) in mice leads to low bone mass and accelerates ovariectomy-associated bone loss by increasing osteoclastogenesis. Bone. Jul 2015;76:107-14.

124. Michalski MN, Seydel AL, Siismets EM, Zweifler LE, Koh AJ, Sinder BP, et al. Inflammatory bone loss associated with MFG-E8 deficiency is rescued by teriparatide. FASEB J. Jul 2018;32(7):3730-41.

125. Li X, Liu H, Qin L, Tamasi J, Bergenstock M, Shapses S, et al. Determination of dual effects of parathyroid hormone on skeletal gene expression in vivo by microarray and network analysis. J Biol Chem. Nov 9 2007;282(45):33086-97.

126. Paavola CD, Hemmerich S, Grunberger D, Polsky I, Bloom A, Freedman R, et al. Monomeric monocyte chemoattractant protein-1 (MCP-1) binds and activates the MCP-1 receptor CCR2B. J Biol Chem. Dec 11 1998;273(50):33157-65.

127. Graves DT, Jiang Y, Valente AJ. Regulated expression of MCP-1 by osteoblastic cells in vitro and in vivo. Histol Histopathol. Oct 1999;14(4):1347-54.

128. Tamasi JA, Vasilov A, Shimizu E, Benton N, Johnson J, Bitel CL, et al. Monocyte chemoattractant protein-1 is a mediator of the anabolic action of parathyroid hormone on bone. J Bone Miner Res. Sep 2013;28(9):1975-86.

129. Chang MK, Raggatt LJ, Alexander KA, Kuliwaba JS, Fazzalari NL, Schroder K, et al. Osteal tissue macrophages are intercalated throughout human and mouse bone lining tissues and regulate osteoblast function in vitro and in vivo. J Immunol. Jul 15 2008;181(2):1232-44.

130. Cho SW, Soki FN, Koh AJ, Eber MR, Entezami P, Park SI, et al. Osteal macrophages support physiologic skeletal remodeling and anabolic actions of parathyroid hormone in bone. Proc Natl Acad Sci U S A. Jan 28 2014;111(4):1545-50.

131. Michalski MN, Koh AJ, Weidner S, Roca H, McCauley LK. Modulation of osteoblastic cell efferocytosis by bone marrow macrophages. J Cell Biochem. Dec 2016;117(12):2697-706.

132. Alford AI, Kozloff KM, Hankenson KD. Extracellular matrix networks in bone remodeling. Int J Biochem Cell Biol. Aug 2015;65:20-31.

133. Bouleftour W, Bouet G, Granito RN, Thomas M, Linossier MT, Vanden-Bossche A, et al. Blocking the expression of both bone sialoprotein (BSP) and osteopontin (OPN) impairs the anabolic action of PTH in mouse calvaria bone. J Cell Physiol. Mar 2015;230(3):568-77.

134. Denhardt DT, Noda M. Osteopontin expression and function: role in bone remodeling. J Cell Biochem. 1998;72 Suppl 30-31(S30-31):92-102.

135. Kitahara K, Ishijima M, Rittling SR, Tsuji K, Kurosawa H, Nifuji A, et al. Osteopontin deficiency induces parathyroid hormone enhancement of cortical bone formation. Endocrinology. May 2003;144(5):2132-40.

136. Bonnet N, Conway SJ, Ferrari SL. Regulation of β catenin signaling and parathyroid hormone anabolic effects in bone by the matricellular protein periostin. Proc Natl Acad Sci U S A. Sep 11 2012;109(37):15048-53.

137. Machado do Reis L, Kessler CB, Adams DJ, Lorenzo J, Jorgetti V, Delany AM. Accentuated osteoclastic response to parathyroid hormone undermines bone mass acquisition in osteonectin-null mice. Bone. Aug 2008;43(2):264-73.

138. Novince CM, Koh AJ, Michalski MN, Marchesan JT, Wang J, Jung Y, et al. Proteoglycan 4, a novel immunomodulatory factor, regulates parathyroid hormone actions on hematopoietic cells. Am J Pathol. Nov 2011;179(5):2431-42.

139. Novince CM, Michalski MN, Koh AJ, Sinder BP, Entezami P, Eber MR, et al. Proteoglycan 4: a dynamic regulator of skeletogenesis and parathyroid hormone skeletal anabolism. J Bone Miner Res. Jan 2012;27(1):11-25.

140. Selvamurugan N, Pulumati MR, Tyson DR, Partridge NC. Parathyroid hormone regulation of the rat collagenase-3 promoter by protein kinase A-dependent transactivation of core binding factor α1. J Biol Chem. Feb 18 2000;275(7):5037-42.

141. Merciris D, Schiltz C, Legoupil N, Marty-Morieux C, de Vernejoul MC, Geoffroy V. Over-expression of TIMP-1 in osteoblasts increases the anabolic response to PTH. Bone. Jan 2007;40(1):75-83.

142. Geoffroy V, Marty-Morieux C, Le Goupil N, Clement-Lacroix P, Terraz C, Frain M, et al. In vivo inhibition of osteoblastic metalloproteinases leads to increased trabecular bone mass. J Bone Miner Res. May 2004;19(5):811-22.

143. Delanote V, Vandekerckhove J, Gettemans J. Plastins: versatile modulators of actin organization in (patho)physiological cellular processes. Acta Pharmacol Sin. Jul 2005;26(7):769-79.

144. van Dijk FS, Zillikens MC, Micha D, Riessland M, Marcelis CL, de Die-Smulders CE, et al. PLS3 mutations in X-linked osteoporosis with fractures. N Engl J Med. Oct 17 2013;369(16):1529-36.

145. Valimaki VV, Makitie O, Pereira R, Laine C, Wesseling-Perry K, Maatta J, et al. Teriparatide treatment in patients with WNT1 or PLS3 mutation-related early-onset osteoporosis: a pilot study. J Clin Endocrinol Metab. Feb 1 2017;102(2):535-44.

146. Yorgan TA, Sari H, Rolvien T, Windhorst S, Failla AV, Kornak U, et al. Mice lacking plastin-3 display a specific defect of cortical bone acquisition. Bone. Jan 2020;130:115062.

147. Huck K, Sens C, Wuerfel C, Zoeller C, Nakchbandi IA. The Rho GTPase RAC1 in osteoblasts controls their function. Int J Mol Sci. Jan 8 2020;21(2).

148. Kawano T, Troiano N, Adams DJ, Wu JJ, Sun BH, Insogna K. The anabolic response to parathyroid hormone is augmented in Rac2 knockout mice. Endocrinology. Aug 2008;149(8):4009-15.

149. Larson CM, Henderson RC. Bone mineral density and fractures in boys with Duchenne muscular dystrophy. J Pediatr Orthop. Jan-Feb 2000;20(1):71-4.

150. Gray SK, McGee-Lawrence ME, Sanders JL, Condon KW, Tsai CJ, Donahue SW. Black bear parathyroid hormone has greater anabolic effects on trabecular bone in dystrophin-deficient mice than in wild type mice. Bone. Sep 2012;51(3):578-85.

151. Pacheco-Costa R, Davis HM, Sorenson C, Hon MC, Hassan I, Reginato RD, et al. Defective cancellous bone structure and abnormal response to PTH in cortical bone of mice lacking Cx43 cytoplasmic C-terminus domain. Bone. Dec 2015;81:632-43.

152. Brown EM, Pollak M, Hebert SC. The extracellular calcium-sensing receptor: its role in health and disease. Annu Rev Med. 1998;49:15-29.

153. Al-Dujaili SA, Koh AJ, Dang M, Mi X, Chang W, Ma PX, et al. Calcium sensing receptor function supports osteoblast survival and acts as a co-factor in PTH anabolic actions in Bone. J Cell Biochem. Jul 2016;117(7):1556-67.

154. Xue Y, Karaplis AC, Hendy GN, Goltzman D, Miao D. Genetic models show that parathyroid hormone and 1,25-dihydroxyvitamin D3 play distinct and synergistic roles in postnatal mineral ion homeostasis and skeletal development. Hum Mol Genet. Jun 1 2005;14(11):1515-28.

155. Samadfam R, Xia Q, Miao D, Hendy GN, Goltzman D. Exogenous PTH and endogenous 1,25-dihydroxyvitamin D are complementary in inducing an anabolic effect on bone. J Bone Miner Res. Aug 2008;23(8):1257-66.

156. Suryadinata R, Sadowski M, Sarcevic B. Control of cell cycle progression by phosphorylation of cyclin-dependent kinase (CDK) substrates. Biosci Rep. Mar 17 2010;30(4):243-55.

157. Takahashi A, Mulati M, Saito M, Numata H, Kobayashi Y, Ochi H, et al. Loss of cyclin-dependent kinase 1 impairs bone formation, but does not affect the bone-anabolic effects of parathyroid hormone. J Biol Chem. Dec 14 2018;293(50):19387-99.

158. Wang Y, Wan C, Deng L, Liu X, Cao X, Gilbert SR, et al. The hypoxia-inducible factor α pathway couples angiogenesis to osteogenesis during skeletal development. J Clin Invest. Jun 2007;117(6):1616-26.

159. Riddle RC, Leslie JM, Gross TS, Clemens TL. Hypoxia-inducible factor-1α protein negatively regulates load-induced bone formation. J Biol Chem. Dec 30 2011;286(52):44449-56.

160. Frey JL, Stonko DP, Faugere MC, Riddle RC. Hypoxia-inducible factor-1α restricts the anabolic actions of parathyroid hormone. Bone Res. 2014;2:14005.

161. Fu Q, Manolagas SC, O'Brien CA. Parathyroid hormone controls receptor activator of NF-kappaB ligand gene expression via a distant transcriptional enhancer. Mol Cell Biol. Sep 2006;26(17):6453-68.

162. Walker EC, Poulton IJ, McGregor NE, Ho PW, Allan EH, Quach JM, et al. Sustained RANKL response to parathyroid hormone in oncostatin M receptor-deficient osteoblasts converts anabolic treatment to a catabolic effect in vivo. J Bone Miner Res. Apr 2012;27(4):902-12.

163. Poli A, Bruschi F, Cesana B, Rossi M, Paoletti R, Crosignani PG. Plasma low-density lipoprotein cholesterol and bone mass densitometry in postmenopausal women. Obstet Gynecol. Nov 2003;102(5 Pt 1):922-6.

164. Huang MS, Lu J, Ivanov Y, Sage AP, Tseng W, Demer LL, et al. Hyperlipidemia impairs osteoanabolic effects of PTH. J Bone Miner Res. Oct 2008;23(10):1672-9.

165. Li X, Garcia J, Lu J, Iriana S, Kalajzic I, Rowe D, et al. Roles of parathyroid hormone (PTH) receptor and reactive oxygen species in hyperlipidemia-induced PTH resistance in preosteoblasts. J Cell Biochem. Jan 2014;115(1):179-88.

166. Elefteriou F, Ahn JD, Takeda S, Starbuck M, Yang X, Liu X, et al. Leptin regulation of bone resorption by the sympathetic nervous system and CART. Nature. Mar 24 2005;434(7032):514-20.

167. Hanyu R, Wehbi VL, Hayata T, Moriya S, Feinstein TN, Ezura Y, et al. Anabolic action of parathyroid hormone regulated by the β2-adrenergic receptor. Proc Natl Acad Sci U S A. May 8 2012;109(19):7433-8.

168. McKelvey KD, Fowler TW, Akel NS, Kelsay JA, Gaddy D, Wenger GR, et al. Low bone turnover and low bone density in a cohort of adults with Down syndrome. Osteoporos Int. Apr 2013;24(4):1333-8.

169. Fowler TW, McKelvey KD, Akel NS, Vander Schilden J, Bacon AW, Bracey JW, et al. Low bone turnover and low BMD in Down syndrome: effect of intermittent PTH treatment. PLoS One. 2012;7(8):e42967.

170. Blackwell KA, Raisz LG, Pilbeam CC. Prostaglandins in bone: bad cop, good cop? Trends Endocrinol Metab. May 2010;21(5):294-301.

171. Xu M, Choudhary S, Voznesensky O, Gao Q, Adams D, Diaz-Doran V, et al. Basal bone phenotype and increased anabolic responses to intermittent parathyroid hormone in healthy male COX-2 knockout mice. Bone. Aug 2010;47(2):341-52.

172. Kriegel AJ, Liu Y, Fang Y, Ding X, Liang M. The miR-29 family: genomics, cell biology, and relevance to renal and cardiovascular injury. Physiol Genomics. Feb 27 2012;44(4):237-44.

173. Hrdlicka HC, Pereira RC, Shin B, Yee SP, Deymier AC, Lee SK, et al. Inhibition of miR-29-3p isoforms via tough decoy suppresses osteoblast function in homeostasis but promotes intermittent parathyroid hormone-induced bone anabolism. Bone. Feb 2021;143:115779.

174. Rowe DW, Adams DJ, Hong SH, Zhang C, Shin DG, Renata Rydzik C, et al. Screening gene knockout mice for variation in bone mass: analysis by µCT and histomorphometry. Curr Osteoporos Rep. Apr 2018;16(2):77-94.
